# Quantitative Histopathology Analysis Based on Label-free Multiphoton Imaging for Breast Cancer Diagnosis and Neoadjuvant Immunotherapy Response Assessment

**DOI:** 10.7150/ijbs.102744

**Published:** 2025-01-01

**Authors:** Ruiqi Zhong, Ying Zhang, Wenzhuo Qiu, Kaipeng Zhang, Qianqian Feng, Xiuxue Cao, Qixin Huang, Yijing Zhang, Yuanyuan Guo, Jia Guo, Lingyu Zhao, Xiuhong Wang, Shuhao Wang, Lifang Cui, Aimin Wang, Haili Qian, Fei Ma

**Affiliations:** 1 4+4 Medical Doctor Program, Chinese Academy of Medical Sciences & Peking Union Medical College, Beijing 100730, China.; 2 Department of Medical Oncology, National Cancer Center/National Clinical Research Center for Cancer/Cancer Hospital, Chinese Academy of Medical Sciences and Peking Union Medical College, Beijing 100021, China.; 3 Academy of Advanced Interdisciplinary Study, Peking University, Beijing 100871, China.; 4 High-Tech Research and Development Center (Administrative Center for Basic Research), National Natural Science Foundation of China, Beijing 100044, China.; 5 Department of Pathology, China-Japan Friendship Hospital, Beijing 100029, China.; 6 College of Future Technology, Peking University, Beijing 100871, China.; 7 Chinese Academy of Medical Sciences & Peking Union Medical College, Beijing 100006, China.; 8 Thorough Lab, Thorough Future, Beijing 100036, China; 9 School of Electronics, Peking University, Beijing 100871, China.; 10 State Key Laboratory of Advanced Optical Communication System and Networks, Peking University, Beijing 100871, China.; 11 State Key Laboratory of Molecular Oncology, National Cancer Center/National Clinical Research Center for Cancer/Cancer Hospital, Chinese Academy of Medical Sciences and Peking Union Medical College, Beijing 100021, China.

**Keywords:** label-free multiphoton imaging, quantitative analysis, breast cancer, neoadjuvant immunotherapy, extracellular matrix, texture

## Abstract

Accurate diagnosis and assessment of breast cancer treatment responses are critical challenges in clinical practice, influencing patient treatment strategies and ultimately long-term prognosis. Currently, diagnosing breast cancer and evaluating the efficacy of neoadjuvant immunotherapy (NAIT) primarily rely on pathological identification of tumor cell morphology, count, and arrangement. However, when tumors are small, the tumors and tumor beds are difficult to detect; relying solely on tumor cell identification may lead to false negatives. In this study, we used the label-free multiphoton microscopy (MPM) method to quantitatively analyze breast tissue at the cellular, extracellular, and textural levels, and identified 11 key factors that can effectively distinguish different types of breast diseases. Key factors and clinical data are used to train a two-stage machine learning automatic diagnosis model, MINT, to accurately diagnose breast cancer. The classification capability of MINT was validated in independent cohorts (stage 1 AUC = 0.92; stage 2 AUC = 1.00). Furthermore, we also found that some factors could predict and assess the efficacy of NAIT, demonstrating the potential of label-free MPM in breast cancer diagnosis and treatment. We envision that in the future, label-free MPM can be used to complement stromal and textural information in pathological tissue, benefiting breast cancer diagnosis and neoadjuvant therapy efficacy prediction, thereby assisting clinicians in formulating personalized treatment plans.

## Introduction

According to the latest released data from the International Agency for Research on Cancer (IARC), part of the World Health Organization (WHO), breast cancer is the second most commonly diagnosed cancer worldwide, following lung cancer, with more than 2.3 million new cases in 2022, seriously threatens women's health (https://gco.iarc.fr/en). Accurate diagnosis and assessment of breast cancer treatment efficacy play pivotal roles in oncological decisions on subsequent treatment plans, chemotherapy regimens, and ultimately influencing the prognosis of patients. Currently, the diagnosis and the assessment of the response to treatment (especially the evaluation of neoadjuvant therapy) mainly rely on the identification of tumor cell morphology, number and arrangement by core needle biopsy or postoperative pathological examination 1-4. However, in some early-stage breast cancer patients or those after neoadjuvant therapy, false-negative results may occur due to the limited distribution of tumor cells and failure to cut into the tumor bed during specimen collection or sectioning 5-7. Consequently, integrating the assessment of additional factors within the tumor microenvironment becomes imperative.

In addition to the cellular and histomorphologic information provided by hematoxylin-eosin staining (H&E) staining, which is the most commonly used for pathologic examination, many techniques can provide additional information about breast disease, such as Masson's trichrome (MT) staining, which can provide information about the extracellular matrix, and Immunohistochemistry (IHC), which can provide information about molecular pathology; however, both take several days to weeks and will sometimes delay the diagnosis 8, 9. Besides, mammography, ultrasound, and magnetic resonance imaging (MRI) are widely utilized clinical modalities for adjunctive diagnosis and treatment response assessment in breast cancer and can provide information on the overall shape of the breast mass. However, these techniques had limitations in both sensitivity and specificity, leading to the development of alternative techniques. To be more specific, mammography has reduced sensitivity in pre-menopausal women because of the higher density of gland-rich breasts, which can produce false negatives 10-12. Ultrasound has limitations in detecting breast lesions smaller than 1 cm in diameter, inability to differentiate between tumors and fibrosis, and the detection of nonpalpable tumors is technique-dependent and time-consuming, which reduces the uniformity of detection 13-16. MRI is too expensive and is not always available in primary hospitals 17. Given these challenges, there is a pressing need for a diagnostic tool that offers more information on the microenvironment, with higher resolution, nontoxic, and faster results, which brings us to the potential of multiphoton imaging (MPM).

MPM is an advanced imaging technology with several advantages, such as low photo-damage, low light-bleaching, cross-section ability, high penetration depth, and the ability to provide subcellular resolution in unstained tissues 16, 18-21. Two-photon excitation fluorescence (TPEF) and second-harmonic generation (SHG) are the most common multiphoton phenomenon utilized in MPM. Specifically, TPEF imaging helps visualize the cellular structure, subcellular details, and the elastic fibers of the extracellular matrix, while SHG imaging, an optically nonlinear coherent process, produces information on collagen fibers 22, 23. Collagen, as a major component of the extracellular matrix, forms the scaffold of the tumor microenvironment and exerts an influence on it 24. It regulates extracellular matrix remodeling through degradation and redeposition, thereby promoting tumor infiltration, angiogenesis, invasion, and migration. Currently, collagen is recognized as a double-edged sword in tumorigenesis and progression. On the one hand, it acts as a passive barrier against tumor cells; on the other hand, extracellular matrix remodeling leads to morphological changes in collagen fibers, which can facilitate the migration of tumor cells 25. This phenomenon has been corroborated in studies of breast cancer and liver cancer 26-28. Therefore, focusing on changes in the extracellular matrix is crucial for researching the diagnosis and treatment of tumors. In addition to that, both TPEF and SHG provide information about the fluorescence intensity reflecting the texture of the tissue. The combination of the three aspects, cells, extracellular matrix, and texture, reveals comprehensive insights into the microenvironment of the breast lesions. Nowadays, the MPM has been widely applied in diagnostic and prognostic prediction studies of breast lesion, confirming that MPM can visualize basement membrane of the duct 9, observe lymph vascular invasion 29, distinguish tumor tissues from benign tissues 30-33, identify the histopathology grade of the invasive carcinoma 34, 35, predict the response of neoadjuvant chemotherapy 16, 36, 37, and predict the risk of metastasis and long-term prognosis 35, 38-45. However, to the best of our knowledge, research on the diagnostic and treatment response assessment potential of quantitative histopathologic analysis of MPM remains limited.

In this study, we applied label-free MPM to perform quantitative analysis of tumor cells, extracellular matrix, and texture. We identified 11 key factors from this analysis and developed a multi-omic two-stage machine learning automatic model, MINT, for a broad spectrum of breast lesions diagnosis, specifically, benign lesions, carcinoma *in situ* (CIS), and invasive carcinoma (IC). In addition, by monitoring these key factors, we evaluated the response of neoadjuvant immunotherapy, anti-PD-1/ anti-PD-L1, and observed that the up-regulation of collagen density and intensity of collagen, the down-regulation of collagen orientation and standard deviation of collagen orientation were related with pathological complete response (pCR) in NAIT. However, the up-regulation of the nucleus area, collagen orientation, standard deviation of collagen orientation, and the down-regulation of elastin density were related to non-pCR in NAIT. Besides, by comparing the baseline level of the quantitative factors, we found out that the high level of average elastin intensity and standard deviation of elastin intensity were related to effective treatment in NAIT.

## Materials and Methods

### Ethics Statement

This multicenter retrospective study has received ethical approval from the Ethics Committee and Institutional Review Boards of the China-Japan Friendship Hospital (2023-KY-287) and Cancer Hospital, Chinese Academy of Medical Sciences and Peking Union Medical College (NCC2023C-819). As this was a retrospective study, the requirement for informed consent was waived.

### Study Participants and Sample Preparation

We collected formalin-fixed paraffin-embedded (FFPE) tissues from 50 patients with breast lesions diagnosed in China-Japan Friendship Hospital (Beijing, China), and both biopsy and surgical FFPE tissues from 21 patients received neoadjuvant immunotherapy in Cancer Hospital, Chinese Academy of Medical Sciences and Peking Union Medical College (Beijing, China). The clinicopathologic characteristics of these two cohorts are shown in Table 1, and detailed information for individual participants is shown in Table S1 and Table S2.

Three consecutive slices (5 μm thickness each) were obtained from each FFPE tissue block. We then deparaffinized the slices with alcohol and xylene. One slice of each block underwent multiphoton imaging, and others were stained by H&E and MT separately for histological examination. All the preparation and staining procedures were conducted in the pathology department at China-Japan Friendship Hospital.

### MPM System

A homemade two-photon microscopy combined with a tunable Ti: sapphire laser (Insight X3+, MKS Instruments, Massachusetts, USA) was utilized to image the samples, with 760 nm chosen as the optimal excitation wavelength in our study. The images were acquired by a galvanometer optical scanner (6215H, Cambridge Technology, Bedford, USA) at 500Hz bidirectionally, achieving two frames per second for a 512×512 pixels image. A water-immersion objective (XLPLN25XWMP2, 25x/1.05 NA, Olympus, Tokyo, Japan) was used, archiving a field of view (FOV) as large as 303×303 μm^2^. The average laser power was 20mW at the focus.

The reflected signals were split through a long-pass dichroic mirror (FF495-Di03-25x36, IDEX Health & Science, New York, USA) and went through a band-pass filter (IDEX Health & Science, New York, USA) separately: 389 ± 38 nm for SHG signals and 475± 50 nm for TPEF signals. Two photomultiplier tubes (H10770PA-40, Hamamatsu Photonics K.K., Hamamatsu City, Japan) captured those signals.

A high-speed motorized XY scanning stage (MLS203, Thorlabs, New Jersey, USA) contributed to the acquisition of large-scale stitched images. To achieve a higher signal-to-noise ratio, each FOV was acquired 5 frames repeatedly. It took about 4 minutes to cover an area of 3 mm×3 mm, enabling rapid, large-scale imaging for FFPE samples.

### Morphological Factors

Regions of suspicious lesions and normal breast tissue adjacent to carcinoma (shown in Table S1) were selected by a qualified pathologist in multiphoton images. We selected 20 cells and 20 ROIs (regions of interest) for each slice randomly. Then, we measured cellular factors in the 20 cells and measured extracellular and textural factors separately in every ROI. Cellular factors included nuclear area, cell area, and nucleus-cytoplasmic ratio. Extracellular factors included elastin density (elastic fiber area/ ROI area), collagen density (collagen fiber area/ ROI area), elastin-collagen density ratio, collagen diameter, and collagen orientation. Specifically, all the extracellular factors were measured and calculated within 20 randomly selected ROI of 512×512 pixels within the suspicious region. For the measurement of collagen orientation, we performed a Fast Fourier Transform (FFT) in SHG channel images, and then used the ImageJ software (version 1.51w) for ellipse fitting, and measured the long (L) and short (S) axes, characterized collagen fiber orientation by 1-S/L, where the value closer to 0 indicates greater disorder in collagen fiber alignment, while the value closer to 1 suggests more homotropic alignment 46-49. The average diameter of collagen was analyzed using the DiameterJ (National Institute of Standards and Technology, USA) plug-in in ImageJ 50. The heterogeneity is represented by standard deviation.

### Texture Extraction

The fluorescence intensity of elastin and collagen was measured by the sum of gray values in the fiber region within every 512×512 pixels ROI. We also calculated the average fluorescence intensity of elastin (fluorescence intensity of elastin/ elastin area) and collagen (fluorescence intensity of collagen/ collagen area) for each slice.

### Machine Learning

The model developed in this study is an automatic classification system for diagnosing breast diseases, with the definitive diagnosis provided by expert pathologists based on H&E-stained slices as a ground truth. A total of 14 input features were utilized in the training process, 11 of which were pivotal features extracted from label-free MPM images. These features were selected for their significant variability across different breast disease types, as detailed in Supplementary Table 3 and Figure 3. The remaining three features were from clinical information, encompassing age, metastasis status, and lymph node involvement. To optimize the automatic diagnosis model, we implemented two strategies: direct triclassification and a two-stage model, specifically, the first stage discerns between tumorous and non-tumorous conditions, while the second stage further classifies *in situ* and invasive carcinomas within the tumor category. We also employed four machine learning algorithms: Decision Tree (DT), Multi-Layer Perceptron (MLP), and Support Random Forest (RF), Support Vector Machine (SVM). Given the limitation of sample size, a 7-fold cross-validation approach was adopted for training and internal validation. The model's efficacy was evaluated using accuracy, precision, recall, F1-score, and specificity. The two-stage MLP algorithm outperformed others in model configuration, which we have named the MINT (Multiphoton Imaging-clinical iNformation Two-stage machine learning model). The machine learning modal was established using Python, version 3.11.4 (https://www.python.org/).

### Statistical Analysis

Statistical analysis was implemented using R, version 4.1.3 (http://www.r-project.org/), and GraphPad Prism, version 9.5.1 (https://www.graphpad.com/). If the data or the log transformation of the data followed a normal distribution, a one-way analysis of variance (ANOVA) was conducted to compare three or more groups, followed by Tukey's multiple comparison test or Dunnett's test for further pairwise comparisons. In cases where the data did not meet the assumption of normality, the Kruskal-Wallis test was employed for comparing three or more groups, with Dunn's multiple comparisons test used for pairwise comparisons within the groups. As for comparisons between two groups, if the data followed a Gaussian distribution, paired t-test or unpaired t test with Welch's correction was used; otherwise, Wilcoxon matched-pairs signed rank test or Mann-Whitney test was applied, for paired or non-paired issues respectively. Significance levels are indicated by asterisks (*P < 0.05, **P < 0.01, ***P < 0.001, ****P < 0.0001).

## Results

### Study design and clinicopathological features of the cohorts

We collected FFPE samples of breast lesions from 50 patients who were diagnosed and conducted biopsies or/and operations in CJFH cohort. The samples were imaged using label-free MPM and quantitative histopathology analysis of the relationship between cellular, extracellular, and textural factors and the histological subtype of breast lesion were performed. Subsequently, we identified pivotal factors and developed a machine learning model for automated breast cancer diagnosis. This model, incorporating essential factors and clinical data, facilitates the differentiation between benign lesions, carcinoma *in situ*, and invasive carcinoma. In addition, we further collected FFPE blocks of biopsy and surgical samples from 21 patients in PUCH cohort who received neoadjuvant immunotherapy (NAIT). We applied label-free MPM to collect images of the samples and extracted information on the dynamic changes of key factors, and validated the potential of these key factors to be used to monitor the efficacy of NAIT, we also found that some of the texture factors could predict patients' response before treatment.

Baseline clinicopathologic information for 71 patients from both cohorts is shown in Table 1. Patients with early-onset breast cancer less than or equal to 50 years of age comprised 40.00% (n = 20) and 42.86% (n = 9) of the two cohorts, and patients elder than 50 years of age comprised 60% (n=30) and 57.14% (n = 12), respectively. The CJFH cohort comprised 5 cases of benign lesions and 45 cases of breast cancer, such as CIS (n = 15, 30%), IC (n = 30, 60%), while PUCH cohort were all IC (n = 21, 100%). In all cohorts, 15 (30%) and 0 cases were Tis, 14 (28%) and 1 (4.76%) were T1, 11 (22%) and 17 (80.95%) were T2, 1 (2.00%) and 3 (14.29%) were T3-4, with lymphatic metastasis observed in 10 (20%) and 18 (85.71%) cases, and distant metastasis was presented in 3 (6%) and 0 cases in two cohorts. More detailed baseline characteristics and sampling information for each patient are presented in Supplementary Table 1 and Supplementary Table 2.

### Morphology and texture features vary in different histological subtypes of breast lesions

**Figure 1:**
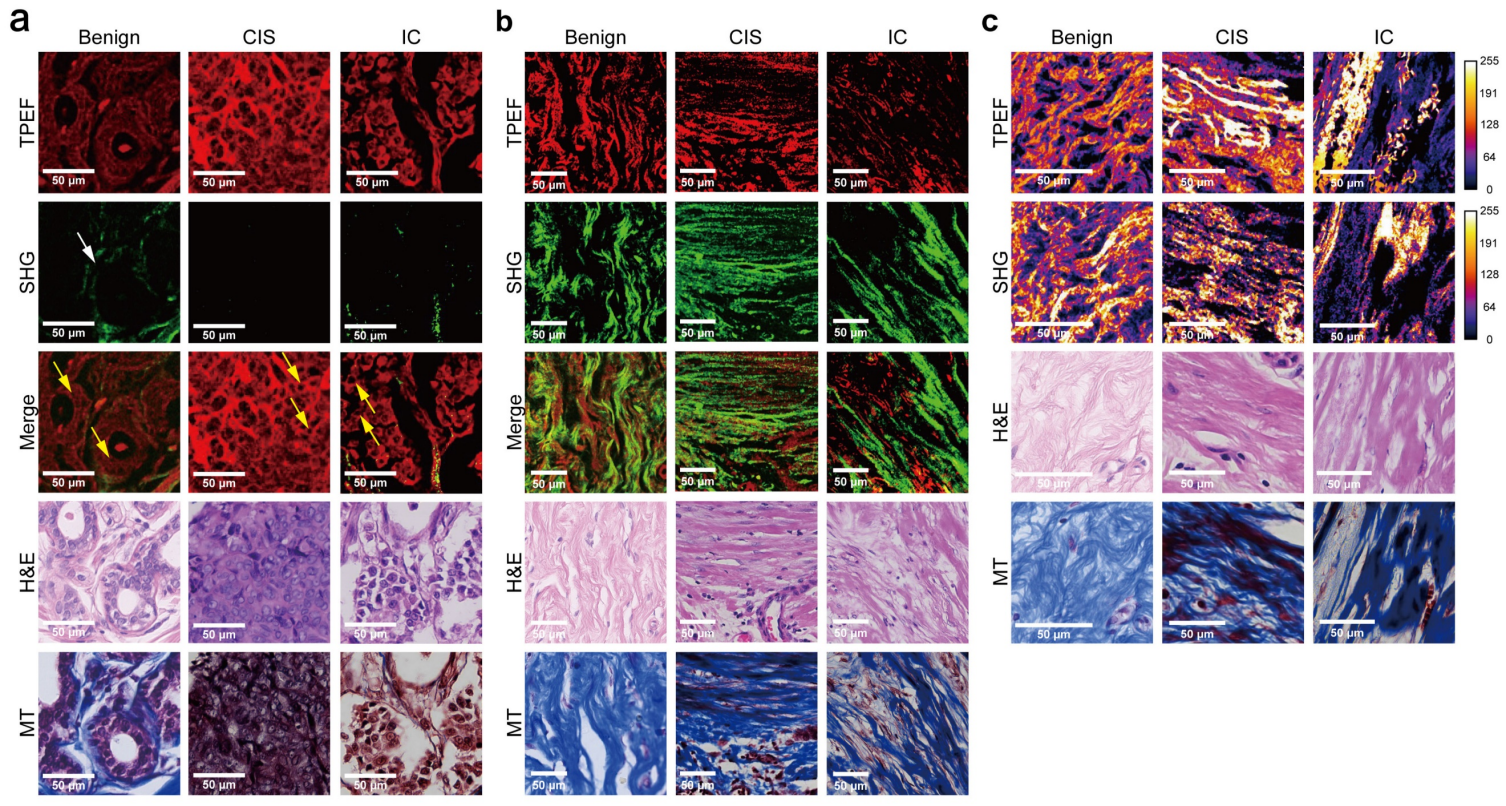
**Comparison of representative label-free MPM images of different breast tissue samples.** Cellular **(a)**, extracellular **(b)** morphology and texture feature **(c)** of benign breast lesion, CIS, and IC. Each column represents the corresponding position of the same tissue. The heatmaps for the TPEF and SHG channels are obtained after converting the images to 8-bit for analysis. Benign, benign breast lesion; Merge, the merge image of TPEF and SHG channel. White arrow: complete basement membrane. Yellow arrow: the black rounded area with no signal represents the nuclei. Scale bar, 50 μm.

One of the aims of this study was to assess the capability of label-free MPM as a diagnostic tool to identify breast lesions in FFPE samples that were unstained. To do this, we first compared the morphological and textural differences in benign breast lesions (including adenosis and peritumoral normal tissues), CIS, and IC. Figure 1 shows representative label-free MPM images of different breast tissue samples and corresponding H&E and MT-stained images. According to previous studies, cells and elastic fibers present auto-fluorescence signals in TPEF channel (red color-coded) and collagen fibers in the extracellular matrix are shown in SHG channel (green color-coded) 32. Specifically, Figure 1a showed that there were two layers of epithelial cells inside the duct in the benign breast lesion: the inner layer consists of ductal epithelial cells, while the outer layer consists of myoepithelial cells. The cells are small, regular, with rounded nuclei (the region inside the cell without signal). However, as for the CIS, the tumor cells with mild atypia proliferated greatly inside the duct and are densely arranged. The nuclei were larger than the one in epithelial cells of the benign lesion, but were still in regular round or ovoid shape. In IC, large and high atypia tumor cells broke through the basement membrane and infiltrated into the stroma. The nuclei of the cells were large and were in irregular shape. Additionally, the differences in the morphological features of the extracellular matrix between different breast tissues are displayed in Figure 1b. To be more specific, the collagen and elastic fibers found within the benign breast lesion exhibited a dense and curved structure, with the basement membranes of the duct and lobule remaining visibly intact (Supplementary Figure 1). However, in contrast to benign tissues, the collagen fibers in CIS exhibited a linear arrangement and decreased diameters. Although the basement membrane remained visible, it appeared significantly enlarged (Supplementary Figure 1). In the case of IC, sparse and linearly arranged collagen fibers of varying diameters proliferated within the stroma. Additionally, there were fractured elastic fibers interspersed among the collagen fibers. The basement membrane disappeared. Due to the small size of Figure 1 images, we showed the TPEF, SHG channels and merge images of benign diseases, CIS and IC, respectively, in larger scopes in Supplementary Figure 2-4 to further demonstrate their differences in cellular, extracellular matrix. Since label-free MPM is based on optical imaging methods, the images have special optical properties that provide texture information as shown in Figure 1c. For benign lesions, the grayscale distribution of TPEF and SHG signals is relatively uniform. As the disease progressed to CIS and IC, the heterogeneity of image grayscale gradually increased. Notably, in IC, distinctive abrupt changes in pixel intensities were evident in both TPEF and SHG signals.

It's worth noting that, label-free MPM images were capable of showing the morphological details of cells and extracellular matrix as the H&E and MT images (Figure 1a-b, Supplementary Figure 1). However, label-free MPM excels at displaying extracellular matrix composition, where collagen fibers produce SHG signals, while elastic and some collagen fibers exhibit autofluorescence. Thus, label-free MPM can effectively differentiate between elastic fibers and collagen fibers within the mesenchyme and present their features independently. Besides, the basement membrane provides SHG signals, which could not be directly observed from the H&E and MT images. Additionally, observations show that the texture information provided by label-free MPM exhibits significant differences in various breast tissues, which cannot be presented by H&E and MT images.

Comparing label-free MPM images of benign breast lesions, CIS, and IC, the description of label-free MPM identifiable features is summarized in Table 2. Label-free MPM can provide valuable additional information from the perspectives of cells, extracellular matrix, and texture, which is meaningful in the diagnosis and differential diagnosis of breast lesions.

### Morphology features of the same patient differ before and after neoadjuvant immunotherapy

**Figure 2:**
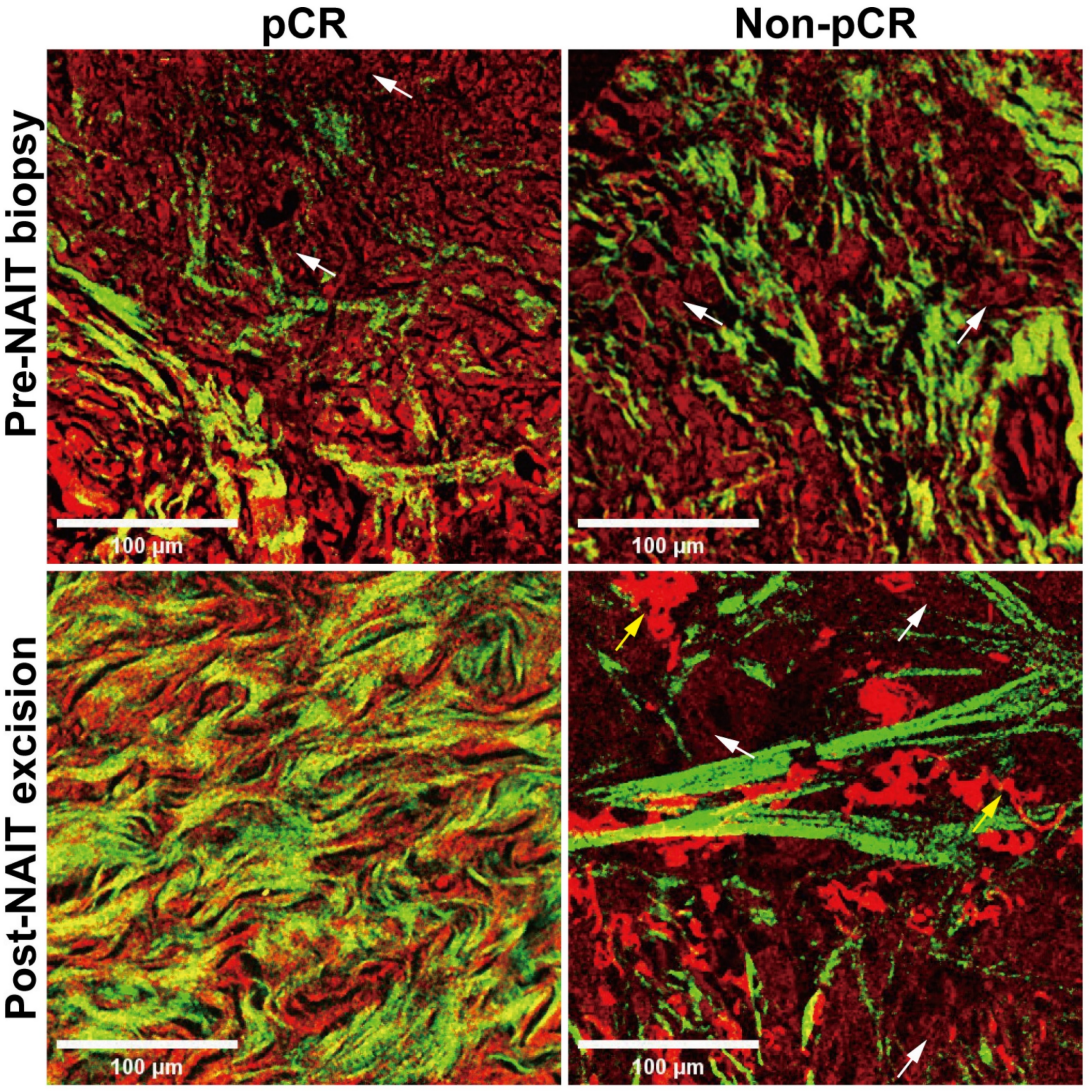
**Morphological changes before and after neoadjuvant immunotherapy in label-free MPM images.** NAIT, neoadjuvant immunotherapy; pCR, pathological complete response. White arrow: tumor cells. Yellow arrow: fractured elastic fibers. Scale bar, 100 μm.

Another object of this study was to explore the potential of label-free MPM in evaluating the efficacy of NAIT. We conducted a comparative analysis using label-free MPM on samples obtained from the same patient before and after NAIT, with representative images of two cases presented in Figure 2. Baseline morphological features were comparable between the two cases before the treatment, with sparse collagen fiber proliferation and tumor cell infiltration in the stroma. However, in the patient who achieved pCR after treatment, tumor cells disappeared, and fibers proliferated significantly in the stroma, primarily characterized by the growth of curly and dense collagen fibers. Conversely, in the non-pCR patient, tumor cells were still present in the stroma after treatment, with collagen fibers maintaining a linear and sparse arrangement. Furthermore, fragmented elastic fibers were notably proliferated in the stroma. These morphological findings illustrate significant differences in label-free MPM imaging between responder and non-responder to NAIT, especially in the changes of the extracellular matrix of pre- and post-treatment. The results suggest the utility of label-free MPM for monitoring treatment efficacy.

### Key cellular, extracellular, and textural factors that are associated with the differential diagnosis of breast cancers

**Figure 3:**
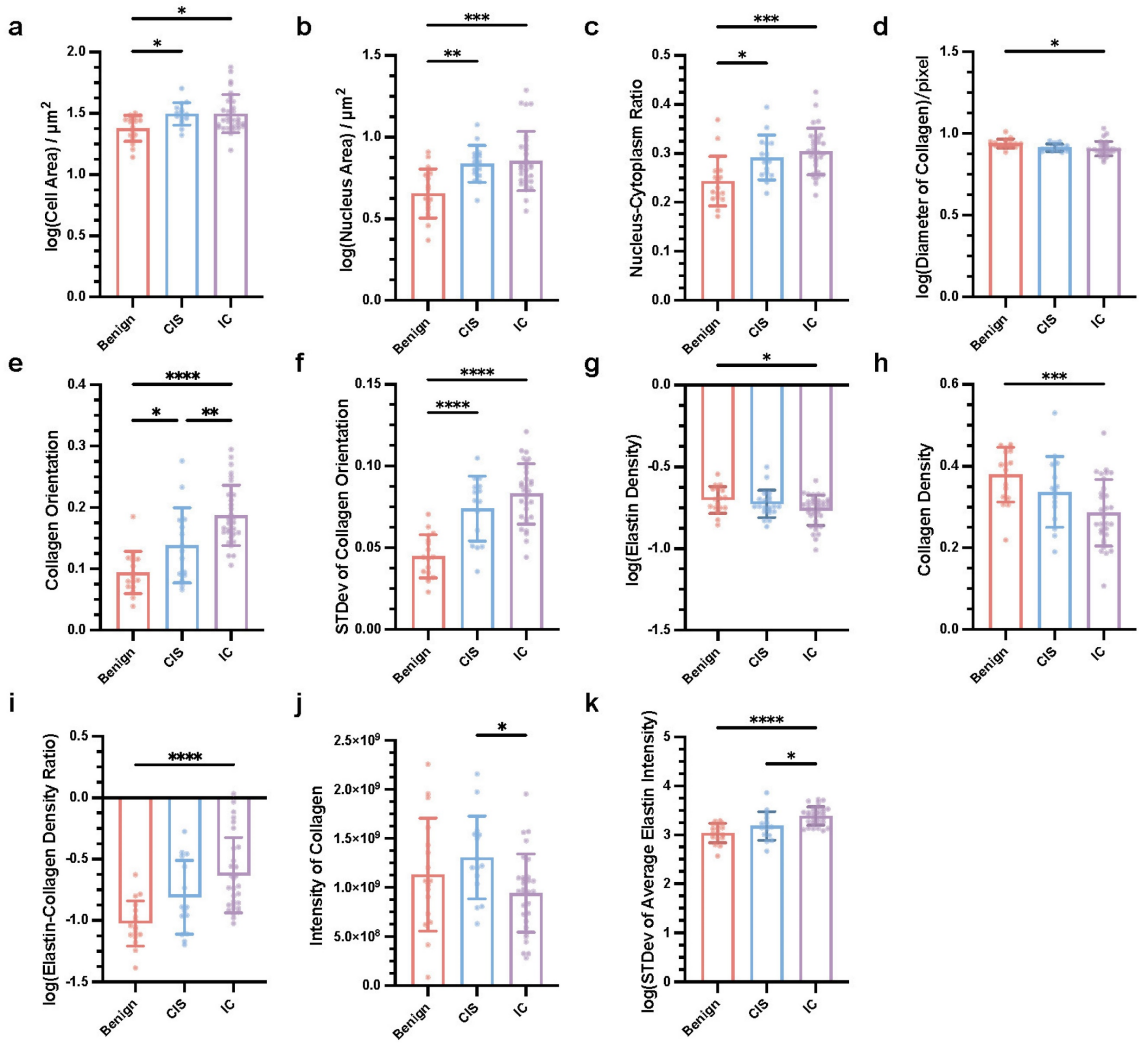
**Key quantitative factors for breast cancer diagnosis in label-free MPM Images.** Mean of cell area **(a)**, nucleus area **(b)**, and nucleus-cytoplasm ratio **(c)**, diameter of collagen **(d)**, collagen orientation **(e)**, elastin density **(g)**, collagen density **(h)**, elastic-collagen density ratio **(i)**, intensity of collagen **(j)**, standard deviation of collagen orientation **(f)** and average elastin intensity were significantly different in three types of breast lesions. n_Benign_=17, n_CIS_=15, n_IC_=30, n refers to the number of slices. STDev, standard deviation. Data are represented as the means ± SD. If the data conforms to a Gaussian distribution, one-way ANOVA with Tukey's test for multiple comparisons in benign breast lesions, CIS and IC; otherwise, Kruskal-Wallis test with Dunn's multiple comparisons test was applied. Significance levels are indicated as follows: *P < 0.05, **P < 0.01, ***P < 0.001, ****P < 0.0001.

As mentioned above, we found that label-free MPM can provide additional information on morphology and texture, aiding in the differential diagnosis of breast cancer. A descriptive summary of the characteristics of label-free MPM for various types of breast diseases was also provided. To further verify the diagnostic ability of label-free MPM, we subsequently designed 24 quantitative features to measure the characteristics of breast cancer in terms of cell, extracellular matrix, and texture (detailed features were presented in Supplementary Table 3). Particularly, to characterize cellular morphology, we based on our prior observations indicating that both cell and nuclear sizes tend to increase with the aggressiveness of the tumor (Table 2). Besides, we were also inspired by the quantitative analyses from previous studies 32, and designed three main features: cell area, nucleus area, and nucleus-cytoplasm ratio to quantitatively describe the cellular morphological features across various breast diseases. Similarly, for characterizing the features of the extracellular matrix, our morphological observations and previous research findings suggest that the stromal composition varies across different breast diseases 9, 32. More specifically, we postulate the existence of fundamental differences in both the content and the ratio of elastic fibers to collagen fibers. To quantify these differences, we devised three features: elastin density, collagen density, and elastin-collagen density ratio. Furthermore, noting variations in collagen fiber morphology, such as curliness and thickness, across different breast diseases, we also adopted additional descriptors, collagen orientation and average collagen diameter to quantitatively describe the characteristics. Furthermore, in terms of texture analysis, prior studies often depict image texture information using intensity 49, 51, 52. Our morphological analysis uncovered notable variations in the optical intensities of TPEF and SHG channels among different breast diseases, underscoring their diagnostic value. Consequently, we identified the total and average optical intensities of elastin and collagen as crucial attributes for our investigation. Noteworthy, in our objective to perform diagnostic assessments leveraging two-photon quantization indicators with individual slices as the basic unit, we computed the mean and standard deviation of these features for each slice to acquire data for subsequent statistical analysis. We followed an interpretable, and reproducible feature extraction pipeline (Supplementary Figure 5) to extract 24 features in every slice (detailed method and formula are shown in Supplementary Table 3). After careful statistical analysis, specifically employing one-way ANOVA with Tukey's test or the Kruskal-Wallis test with Dunn's multiple comparisons, we observed that 11 key factors showed significant differences between breast lesions, as shown in Figure 3. At the cellular level, the mean of cell area, mean of nucleus area, and mean of nucleus-cytoplasm ratio were influential in the differential diagnosis (Figure 3a-c). Specifically, our analysis demonstrated that the cell area, nucleus area, and nucleus-to-cytoplasm ratio in CIS and IC were notably elevated in comparison to Benign lesions. Concerning the extracellular matrix, factors such as mean of collagen diameter, mean of collagen orientation, standard deviation of collagen orientation, mean of elastin density, mean of collagen density, and mean of elastic-collagen density ratio had diagnostic significance. More precisely, the elastin-collagen density in IC was significantly higher than that in benign lesions. Conversely, the diameter of collagen, elastin density, and collagen density were markedly lower in IC compared to benign lesions. Collagen orientation emerged as a notable discriminator among the three conditions, with its value significantly increasing as the disease progressed from Benign to CIS and further to IC. Additionally, we observed that the variability in collagen orientation was significantly greater in both CIS and IC when compared to benign, as evidenced by the higher standard deviations. Furthermore, in terms of texture which was analyzed in 16-bit images, indicators like mean of collagen intensity and standard deviation of average elastin intensity had diagnostic significance. The intensity of collagen in IC was significantly higher than that in CIS. Besides, in terms of the mean elastin fiber intensity, both IC and CIS exhibited significantly higher standard deviations.

In conclusion, we identified 11 key quantitative factors of cells, extracellular matrix, and texture in label-free MPM images that are crucial for the diagnosis and differential diagnosis of breast cancer.

### Establishment and validation of a machine learning model for automatic breast cancer diagnosis based on key factors and clinical information

Next, we attempted to establish an automatic diagnosis model for breast diseases. In addition to the key factors derived from the label-free MPM images, clinical features were also exploited for every patient. In the CJFH cohort, age, nodal status and metastasis status determined by clinical imaging methods were available. Specifically, for patients without imaging-based lymph node status, the postoperative pathological lymph node status was used as a substitute. However, if the postoperative paraffin pathology still did not provide lymph node results for a patient, we regarded it as a negative result. For patients without imaging-based metastasis status, a negative result was also filled in.

**Figure 4:**
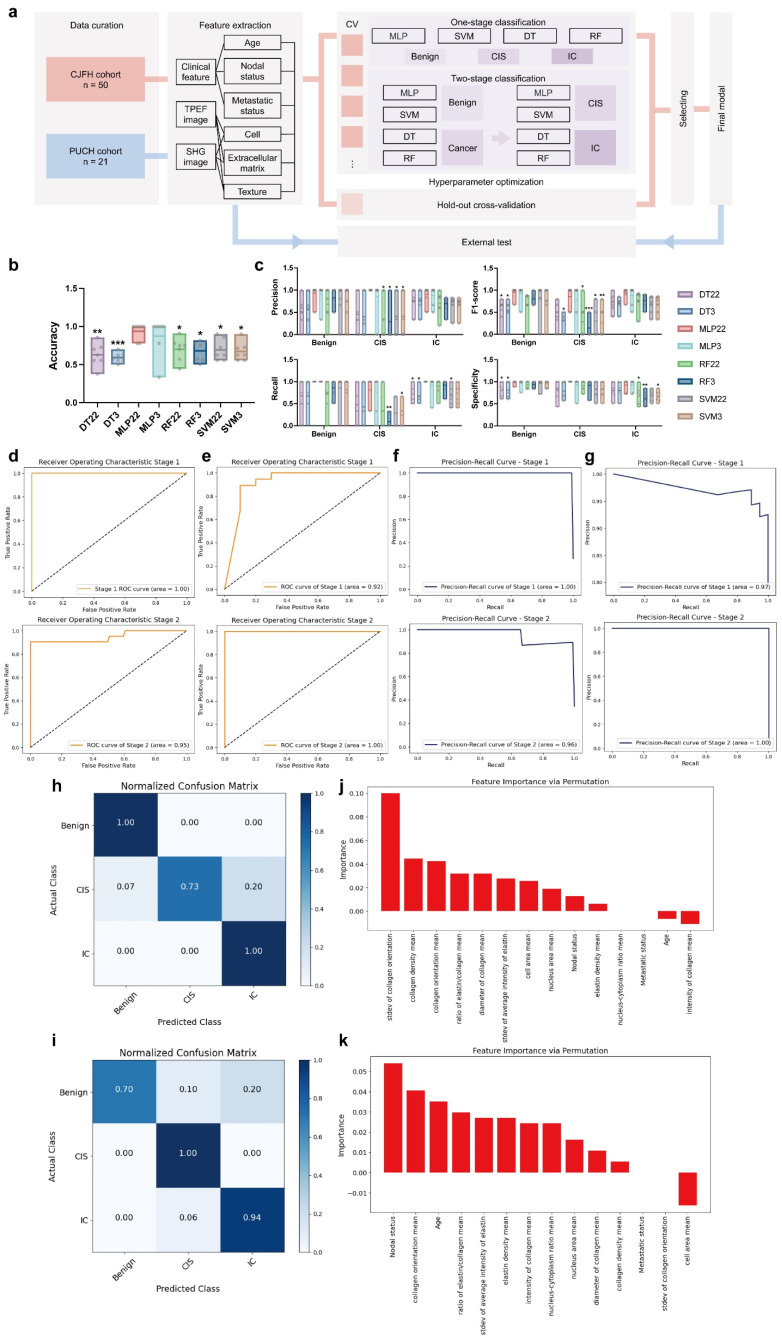
** Training, cross-validation and testing of MINT. (a)** Schematic of the machine learning framework. CV, cross validation; DT, decision-tree; SVM, support vector machine; RF random forest; MLP, multi-layer perceptron. Distribution of each modal's accuracy **(b)**, precision, recall, F1-score, specificity **(c)**. n=7 models were trained during 7-fold cross-validation per group. One-way ANOVA with Dunnett's test was used to carry out multiple comparisons test of MLP22 versus other models. Significance levels are indicated as follows: *P < 0.05, **P < 0.01, ***P < 0.001, ****P < 0.0001. The minimum, maximum, mean, as well as each sample point, are shown. Receiver operating characteristics curves of the CJFH cohort **(d)** and PUCH cohort **(e)**. As for CJFH cohort, the mean of 7-fold cross-validation is shown. Precision-recall curves of the CJFH cohort **(f)** and PUCH cohort **(g)**. As for CJFH cohort, the mean of 7-fold cross-validation is shown. Confusion matrices of CJFH cohort **(h)** and PUCH cohort **(i)** showing the predictions and actual classification of the slices. Data is shown in normalized version. Permutation importance analysis of stage 1 **(j)** and stage 2 (h). The importance of the features decreases from left to right.

We adopted two distinct strategies to construct the model. The first strategy was direct tri-classification, aiming to distinguish between benign breast lesions, CIS, and IC simultaneously. The second strategy adopted a two-stage approach: in the first stage, the model determined whether a sample belonged to a neoplastic disease; subsequently, within the neoplastic samples, the model further discriminated between CIS and IC. The specific workflow for model construction is illustrated in Figure 4a. As for the algorithmic aspects, we applied various machine learning methods, including Decision Tree (DT), Multi-Layer Perceptron (MLP), and Support Random Forest (RF), Support Vector Machine (SVM), to build our models.

We used a 7-fold cross-validation strategy and grouped multiple slices from the same patient into the same group. We then compared the performance of these models using different modeling strategies, as shown in Figure 4b&c. To be more specific, upon 7-fold cross-validation, the accuracy of these models for diagnosis was 62.79% (± 5.82%) for 2-stage decision-tree model (DT22), 59.20% (± 2.50%) for decision-tree model for direct triple classification (DT3), 93.65% (± 4.10%) for 2-stage multi-layer perceptron model (MLP22), 87.30% (± 9.52%) for multi-layer perceptron model for direct triple classification (MLP3), 70.04% (± 5.70%) for 2-stage random forest model (RF22), 68.18% (± 5.06%) for random forest model for direct triple classification (RF3), 69.22% (± 5.30%) for 2-stage support vector machine model (SVM22) and 67.18% (± 4.59%) for support vector machine for direct triple classification (SVM 3). Besides, the specificity for benign breast lesion, CIS, IC was 80.60% (± 6.21%), 82.18% (± 8.10%) and 80.31% (± 6.60%) for DT22; 81.19% (± 6.85%), 78.71% (± 7.73%) and 80.31% (± 6.60%) for DT3; 98.21% (± 1.79%), 100.00% (± 0.00%) and 92.35% (± 4.96%) for MLP22; 96.43% (± 3.57%), 92.86% (± 7.14%) and 92.35% (± 4.96%) for MLP3; 93.45% (± 3.13%), 91.50% (± 4.34%) and 67.04% (± 7.72%) for RF22; 93.45% (± 3.13%), 91.84% (± 6.12%) and 61.46% (± 5.79%) for RF3; 95.92% (± 4.08%), 82.31% (± 7.27%) and 69.93% (± 4.81%) for SVM22; 95.58% (± 2.87%), 82.31% (± 7.27%) and 67.07% (± 4.66%) for SVM 3. The average precision, recall, and F1-score are shown in Table 3 and Figure 4c. Through comparison and analysis using one-way ANOVA with Dunnett's test, we determined that MLP22 outperformed other models in accuracy, precision, recall, F1-score, and specificity. Consequently, we implemented MLP22 algorithms to train a Multiphoton Imaging-clinical iNformation Two-stage machine learning model (MINT). The area under the receiver operating characteristic curve (AUROC) is 1.00 for stage 1 and 0.95 for stage 2 (Figure 4d). Additionally, the area under the precision-recall curve (AUPRC) is 1.00 for stage 1 and 0.96 for stage 2 (Figure 4f). The confusion matrix is shown in Figure 4h.

Next, we assessed whether and how MINT could be applied to a real-life scenario using an independent external test set, which was comprised of a total of 47 slides from 21 breast cancer patients who received NAIT in the PUCH cohort. Specifically, the pre-treatment slides were biopsy samples, while the post-treatment slides were surgical excision samples, and we applied the expert pathologists' diagnosis to determine the ground truth of the histological subtypes. The performance of MINT is shown in Figure 4e&g. The AUROC is 0.92 for stage 1 and 1.00 for stage 2 (Figure 4e). Additionally, the AUPRC is 0.97 for stage 1 and 1.00 for stage 2 (Figure 4g). The accuracy was 89.36%. Besides, the precision was 100.00%, 25.00%, 94.44%; the recall was 70.00%, 100.00%, 94.44%; the F1-score was 82.35%, 40.00%, 94.44%; the specificity was 100.00%, 93.48% and 81.82% for benign breast lesion, CIS, and IC, respectively. The confusion matrix is shown in Figure 4i.

Then, to systematically evaluate the pivotal factors contributing to MINT, we employed the permutation importance method to rank the significance of individual attributes. Notably, within stage 1, the standard deviation of collagen orientation, mean of collagen density, and mean of collagen orientation emerged as key discriminators, effectively distinguishing between tumorous and non-tumorous conditions (Figure 4j). As for stage 2, we observed that nodal status, alongside the mean of collagen orientation and age, played a crucial role in differentiating between CIS and IC (Figure 4k).

### Dynamic changes in the key factors can be applied to monitor the efficacy of neoadjuvant immunotherapies

We have also investigated the dynamic changes in key factors obtained from label-free MPM imaging to evaluate the effectiveness of NAIT in breast cancer patients. Figure 3 revealed intriguing correlations between the values of some key factors and disease progression, ranging from benign conditions to CIS, and ultimately IC. Specifically, consistent upward trends were observed in the mean of nucleus area, mean of collagen orientation, standard deviation of collagen orientation, mean of elastin-collagen density ratio, and standard deviation of average elastin intensity as the disease advanced. Conversely, the mean of elastin density and mean of collagen density displayed a declining trend, indicating alterations in the extracellular matrix composition during tumor progression.

**Figure 5:**
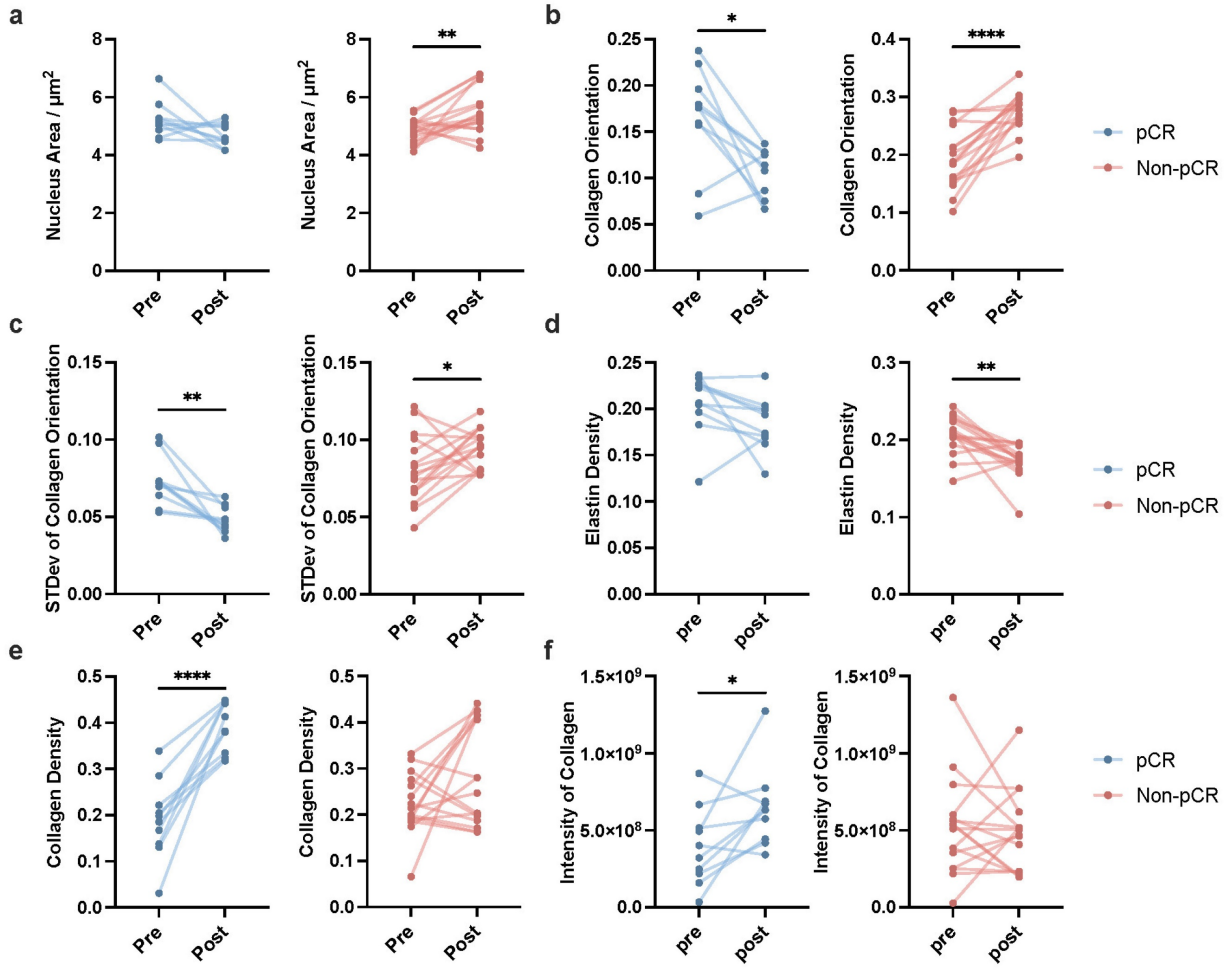
** Dynamic changes in key label-free MPM factors pre- and post-neoadjuvant immunotherapy in patients with breast cancer.** Dynamic changes of mean of nucleus area **(a)**, mean of collagen orientation **(b)**, standard deviation of collagen orientation **(c)**, mean of elastin density **(d)**, mean of collagen density **(e)**, mean of intensity of collagen **(f)** pre- and post-NAIT in breast cancer patients, stratified by pCR or non-pCR. The blue points indicated the dynamic changes of patients who achieved pCR in NAIT; the red points indicated the dynamic changes of patients who did not achieve pCR in NAIT. n_pCR_=10, n_Non-pCR_=16, n refers to the number of slices. Outliers were removed using the ROUT method with an aggressive Q=1%. If the data followed a Gaussian distribution, paired t-test was used to analyze the significance of dynamic changes of each factor pre- and post-treatment; otherwise, Wilcoxon matched-pairs signed rank test was applied. Significance levels are indicated as follows: *P < 0.05, **P < 0.01, ***P < 0.001, ****P < 0.0001.

To further validate the clinical utility of these biomarkers, we conducted a statistical analysis on the key factors obtained from pre- and post-treatment label-free MPM imaging of 21 breast cancer patients undergoing NAIT in the PUCH cohort. As shown in Figure 5, conspicuous distinctions emerged between patients who achieved pCR (represented in blue) and those who did not (represented in red). In terms of nuclear area, patients achieving pCR showed no significant change between pre- and post-treatment, while those who did not reach pCR experienced a significant increase (Figure 5a). The mean of collagen orientation presented a notable decrease in pCR patients, indicating a potential reorganization of the collagen network in response to effective treatment. In contrast, patients who failed to achieve pCR demonstrated a significant increase in the mean of collagen orientation, suggesting continued disease aggressiveness (Figure 5b). Similarly, a consistent trend was observed in the standard deviation of collagen orientation, with a marked reduction in pCR patients and a significant elevation in non-pCR patients, underscoring the potential of this parameter as a treatment outcome indicator (Figure 5c).

Intriguingly, our analysis also revealed distinct changes in the extracellular composition between the two groups. Specifically, patients who achieved pCR exhibited a pronounced increase in fibrous tissue, predominantly collagen fibers (Figure 5e). Conversely, those who did not reach pCR showed a marked decrease in interstitial fibers, with a predominance of elastin loss (Figure 5d). Additionally, while patients achieving pCR demonstrated a significant increase in collagen fiber intensity post-treatment, this enhancement was not evident in non-pCR patients (Figure 5f). The dynamic changes of other key factors are shown in supplementary Figure 6.

### Predictive value of the key factors for neoadjuvant immunotherapeutic response

**Figure 6:**
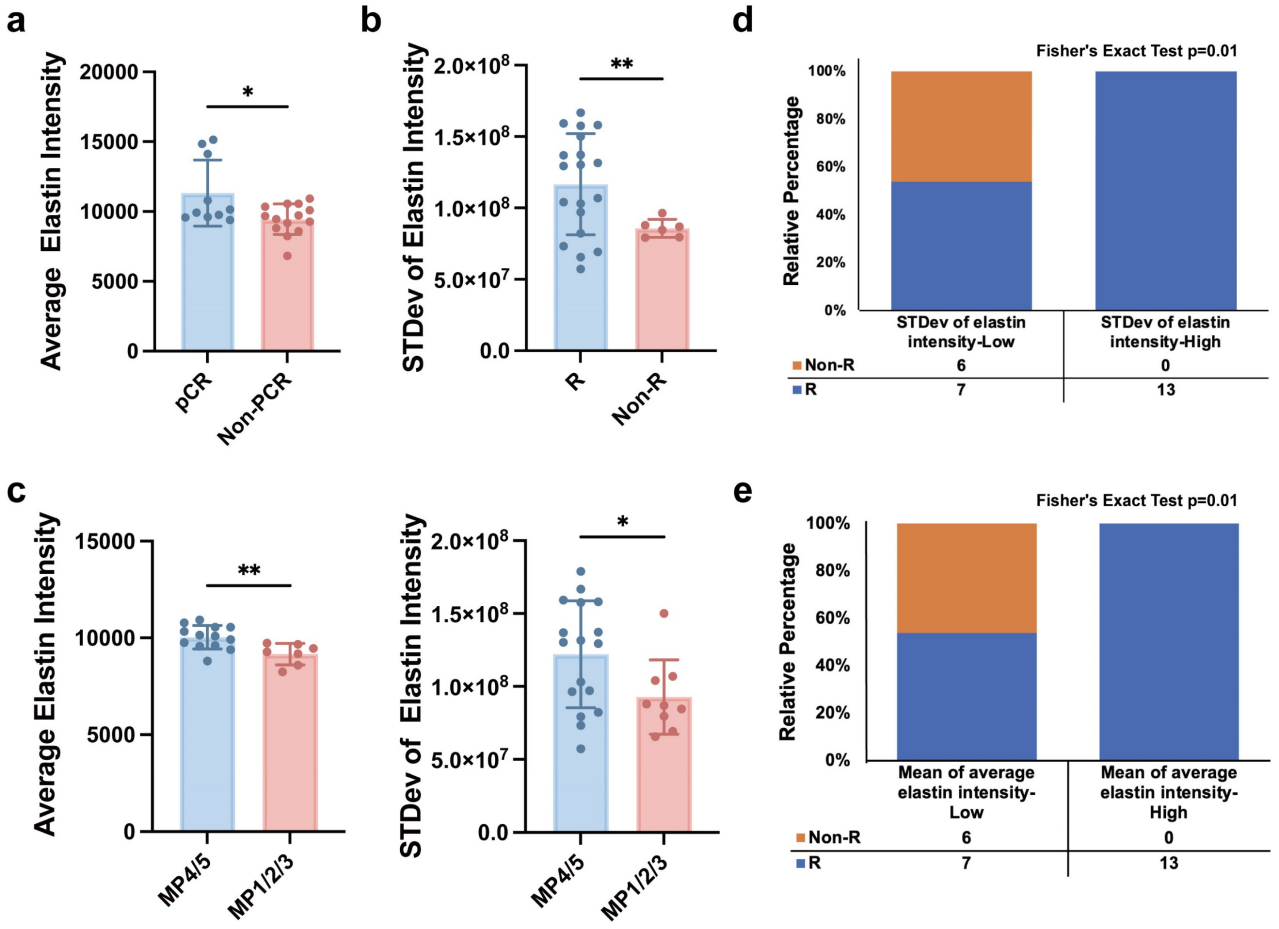
** Label-free MPM factors predict the efficacy of neoadjuvant immunotherapy. (a)** The levels of mean of average elastin intensity were significantly different between pCR and non-pCR patients. **(b)** The levels of standard deviation of elastin intensity were significantly different between responders and non-responders. R, responder; Non-R, non-responder. **(c)** The levels of mean of average elastin intensity and standard deviation of elastin intensity were significantly different between patients with MP grade 4 or 5 and patients with MP grade 1 to 3. **(d&e)** Correlation analysis of the baseline level of standard deviation of elastin intensity or mean of average elastin intensity, and responses of patient with breast cancer to NAIT. R, responder, including patients diagnosed with a CR (complete response) and PR (partial response) after treatment; Non-R, non-responder, including patients diagnosed with an SD (stable disease) and PD (progressive disease) after treatment. n_pCR_=10, n_Non-pCR_=16, n_MP4/5_=17, n_MP1/2/3_=9, n_R_=20, n_Non-R_=6, n refers to the number of slices. Outliers were removed using the ROUT method with an aggressive Q=1%. Data are represented as the means ± SD. If the data followed a Gaussian distribution, unpaired t test with Welch's correction was used to compare the difference between two groups; otherwise, Mann-Whitney test was applied. Significance levels are indicated as follows: *P < 0.05, **P < 0.01, ***P < 0.001, ****P < 0.0001.

We further explored the potential of label-free MPM key factors in predicting the efficacy of NAIT. Three endpoints were included: achievement of pCR, effectiveness assessed by imaging methods (ultrasound or mammography), and the grade of the Miller-Payne grading system. Patients were considered to have responded effectively to NAIT if they achieved pCR, were assessed as having complete response (CR) or partial response (PR) by imaging, or had a Miller-Payne grade of 4 or 5. Comparative analysis of label-free MPM baseline factors in patients before NAIT treatment revealed significant differences in mean of average elastin intensity between pCR and non-pCR groups, as well as between MP4/5 and MP1/2/3 populations. Similarly, significant differences in the stevd of elastin intensity were observed between responder (R) and non-responder (Non-R), as well as between MP4/5 and MP1/2/3 populations, as shown in Figure 6 a-c. These findings demonstrate that label-free MPM factors can effectively predict patient response to NAIT before treatment.

Furthermore, patients were categorized into low and high levels based on the median values of standard deviation of elastin intensity and mean of average elastin intensity. The results showed that patients with high levels of standard deviation of elastin intensity had significantly higher response rates to NAIT than those with low levels, and similar results were observed for mean of average elastin intensity, as illustrated in Figure 5 d&e. We also evaluated the performance of the two patient groups in other treatment endpoints, as presented in Supplementary Figure 7. These findings highlight the potential clinical utility of label-free MPM factors as predictive biomarkers for NAIT response.

## Discussion

Breast cancer is a prevalent cancer among women, and the accurate diagnosis of the disease and assessment of the patient's treatment outcomes are crucial determinants that significantly impact the overall prognosis of the patient. In recent years, NAIT has been gradually applied to patients with locally advanced or inoperable breast cancer, especially in TNBC. However, some patients may not derive benefits from NAIT and could potentially benefit more from alternative treatments like chemotherapy and immediate tumor resection 53, 54. Predicting the response at the baseline stage and monitoring patients' responses to NAIT could substantially reduce overtreatment, alleviate side effects and costs, and optimize individualized treatment plans for patients.

Breast cancer is a heterogeneous tumor consisting of tumor cells as well as stromal cells, immune cells, fibrous tissues, soluble cytokines, etc., that together form the tumor microenvironment. The tumor microenvironment is dynamically changing, responding to and influencing tumor cell behavior, and this relationship is integral to tumor resistance and development 55-58. Currently, pathological diagnosis mainly relies on H&E-stained slices to diagnose patients' diseases and assess the efficacy of immunotherapy by observing the morphology, number, and arrangement of tumor cells. For some patients with smaller tumors or significant tumor shrinkage after neoadjuvant therapy, the tumors and tumor beds are difficult to detect, which may result in false negativity in diagnosis and efficacy assessment. Therefore, we sought to investigate whether the microenvironmental information, such as interstitial fibers and texture provided by label-free MPM imaging is helpful for the diagnosis of breast cancer and the assessment and prediction of the efficacy of NAIT.

In this study, we performed quantitative pathological analysis based on label-free MPM to provide a new perspective for the diagnosis of breast cancer. The results showed that the 11 key quantitative pathological factors could effectively distinguish benign lesions, CIS, and IC in diagnosis. Our study demonstrates that tumor cells exhibit enlarged cellular and nuclear areas, along with a higher nucleus-cytoplasm ratio compared to benign lesions. These findings can be attributed to key characteristics of cancer, including dysregulated cell cycle control, altered gene expression, and metabolic reprogramming affecting nutrient availability 59, 60. Tumor cells often have mutations in cell cycle regulators like RB and TP53, causing uncontrolled proliferation. Missing these controls can lead to increased cell size during cell cycle progression 61. Also, gene expression alterations in tumors can cause overexpression of proliferation genes and downregulation of size control genes, affecting cell and nuclear size 62. Favoring glycolysis in tumors despite oxygen availability, supports rapid cell division and might contribute to larger cell size 63. Besides, we observed a significant reduction in collagen and elastin density within the tumor tissue, concomitant with an increased linearity of collagen fibers. These observations can be attributed to cancer hallmarks such as extracellular matrix remodeling and mechanical stress. Specifically, the tumor microenvironment is marked by dynamic extracellular matrix restructuring. Tumor and stromal cells secrete matrix metalloproteinases and other proteases that degrade the extracellular matrix, including collagen and elastic fibers, to promote tumor invasion and angiogenesis 64-66. The reduced collagen and elastic fiber density may indicate a disrupted balance between matrix synthesis and degradation, with degradation predominating in breast cancer. Additionally, the remodeling process and the increased cellularity and disorganized architecture of tumors can impose mechanical stress on the extracellular matrix, potentially straightening collagen fibers 67. The increased linearity of collagen in CIS and IC has been linked to increased tissue stiffness, which is proven to be related to poor patient prognosis and cancer aggressiveness 68-70. Our study found a sequential increase in collagen linearity across benign diseases, CIS, and IC, correlating with the escalating aggressiveness of breast cancer. Subsequently, building upon the aforementioned 11 key factors, we integrated three prevalent clinical parameters in breast diseases, specifically, age, radiological lymph node status, and metastasis status, to develop a two-stage machine learning diagnostic model, MINT, which showed appealing prediction performance. As far as we know, MINT is currently the first machine learning model capable of assisting in the diagnosis of different breast lesions of various malignancies, demonstrating great innovation and generalizability. The MINT model offers a novel approach to breast cancer diagnosis and strives for precise diagnostic tools in the era of advanced medicine.

In the current study, we also found out that the patient's response to NAIT could be evaluated by monitoring the dynamic changes of some of the key indicators, including nucleus area, collagen orientation, the intensity of collagen, and the density of fibers. Prior studies have shown that collagen density increases after neoadjuvant chemotherapy, which is also related to the response degree of the treatment 37, 71. However, research of utilizing MPM to explore the impact of immunotherapy on breast cancer morphological characteristics remains limited. Neoadjuvant chemotherapy and NAIT operate on different biological mechanisms: the former delivers cytotoxic drugs to tumor cells via the bloodstream to destroy them, while the latter stimulates the body's immune system to promote the infiltration of killer immune cells (such as CD8+ T cells) into the tumor, thereby achieving tumor destruction. Prior studies suggested that the state of the extracellular matrix may influence immune cell infiltration 72, 73. Particularly, we found that the proliferation and decrease of collagen fibers and elastic fibers were not synchronized during the NAIT, with the increase of collagen fibers in patients who achieved pCR, and the decrease of elastic fibers in patients who did not achieve pCR, which may indicate that they play different biological roles in the development of tumors. According to previous studies, alterations in the extracellular matrix can be used as a biomarker of invasion 20, 74-76. However, studies have shown that the role of the extracellular matrix in the process of tumor invasion appears to be ambivalent, with some studies suggesting that it serves as a physical barrier, impeding tumor invasion 77, 78, while other studies have found that it aids the invasion of the tumor through the secretion of cytokines and enzymes, regulation of mesenchymal stiffness 79-81, and being a scaffold for tumor metastasis 74, 82. Our results further suggest that the roles of elastin and collagen may be distinct, further mechanistic studies are needed to explore their specific functions in the initiation, progression, metastasis, and treatment of breast cancer. Besides, we found that the mean of average elastin intensity and the standard deviation of elastin intensity can effectively predict patients' treatment response to NAIT, which is important for realizing personalized medicine and improving patients' treatment plans. These results suggest that cellular, extracellular matrix, and texture information provided by label-free MPM can play an important role in the diagnosis and treatment of breast cancer.

**Figure 7:**
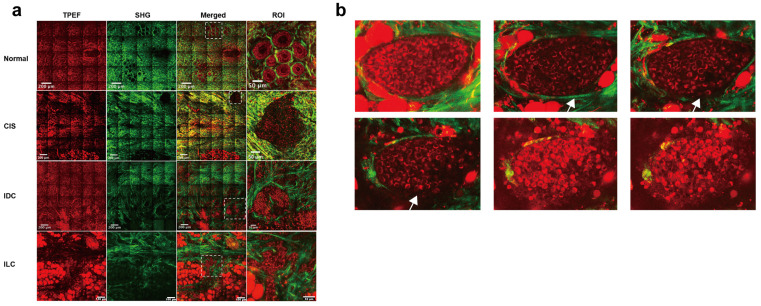
** Label-free MPM imaging in fresh breast tissues. (a)** Representative Label-free MPM images of fresh breast tissue. The dashed rectangle highlights diagnostically significant structures in different breast diseases. Specifically, normal tissue exhibited intact lobular architecture, whereas CIS displays tumor tissue fully encapsulated by collagen fibers. However, tumor cells in IDC and ILC infiltrated into the surrounding stroma. The grid-like lines in the image are caused by the “screen door effect” in image stitching in large-field MPM imaging. **(b)** Stereoscopic imaging of microinvasive breast cancer, with adjacent images spaced 5-10 μm apart along the z-axis. White arrows indicate the progression of the basement membrane from intact to disintegration. IDC, invasive ductal carcinoma; ILC, invasive lobular carcinoma.

We have explored the application of label-free MPM in the diagnosis and treatment of FFPE slices from breast cancer patients. However, one of the notable advantages of label-free MPM is its reduced phototoxicity, making it suitable for *in vivo* imaging 34. It is expected that in the future, the combination of label-free MPM with endoscopic or *in vivo* techniques will enhance the identification, diagnosis, margin determination, and efficacy assessment of breast tumors during surgery and *in vivo* detection 83. Moreover, we conducted an initial exploration into the prospective application of label-free MPM in fresh breast tissues. Immediate imaging was conducted on fresh, unfixed, unsliced, and unstained breast tissues within 30 minutes of surgical excision. This approach revealed the capacity of label-free MPM to delineate the characteristic structures of breast cancer in fresh tissues, as shown in Figure 7a. Notably, fresh tissue imaging provided a stronger TPEF and SHG signal than FFPE samples, which might facilitate intraoperative diagnosis and margin determination in the future.

Furthermore, stereoscopic imaging of the fresh tissue block allowed for comprehensive visualization of microinvasive carcinoma from multiple planes. For example, in Figure 7b, the intact basement membrane of a patient's carcinoma *in situ* was observed in higher planes, while lower planes showed membrane disruption and tumor cell invasion into the mesenchymal stroma, indicative of early micro-invasive carcinoma. However, the diagnosis of microinvasive carcinoma remains difficult in clinical pathology based on slices. These initial findings demonstrate the potential and advantages of label-free MPM, and also pave the way for future *in vivo* studies utilizing label-free MPM.

Despite the positive results of this study, we also recognize its limitations. Firstly, the relatively small sample size of the study and the imbalance between benign breast lesions, CIS and IC in the PUCH cohort, may affect the generalizability of the results. Secondly, the quantitative analysis of cells relied on subjective identification by researchers, introducing potential errors. Thirdly, according to the texture analysis, it commonly includes two aspects, one is fluorescence intensity and the other is the spatial arrangement of the fluorescence. However, the study on the spatial distribution of fluorescence intensity is indeed insufficient in this paper. Fourthly, this study is very preliminary in exploring the application of label-free MPM in fresh tissues and even *in vivo* tissues, and further exploration is needed in the future to evaluate the effectiveness and safety of label-free MPM in fresh tissues. Lastly, the study was retrospective, thereby susceptible to selection and information biases. Future prospective studies are essential to validate our results and extend the applicability of the label-free MPM technique across diverse patient populations.

## Supplementary Material

Supplementary figures and tables.

## Figures and Tables

**Table 1:** Characteristics of patients with breast lesion in multicenter study.

Clinical characteristics	CJFH cohort	PUCH cohort
Total	50	21
**Age, n (%)**		
≤ 50	20 (40.00)	9 (42.86)
>50	30 (60.00)	12 (57.14)
**Sex, n (%)**		
Male	0 (0.00)	0 (0.00)
Female	50 (100.00)	21 (100.00)
**Histological subtype, n (%)**		
Benign	5 (10.00)	0 (0.00)
CIS	15 (30.00)	0 (0.00)
IC	30 (60.00)	21 (100.00)
**Molecular subtype, n (%)**		
Luminal A	13 (26.00)	0 (0.00)
Luminal B	24 (48.00)	3 (14.29)
HER2-enriched	3 (6.00)	0 (0.00)
Triple-negative	4 (8.00)	18 (85.71)
Clinical T stage, n (%)*		
Tis	15 (30.00)	0 (0.00)
T1	14 (28.00)	1 (4.76)
T2	11 (22.00)	17 (80.95)
T3	0 (0.00)	2 (9.52)
T4	1 (2.00)	1 (4.76)
Clinical N stage, n (%)*		
N0	31 (62.00)	3 (14.29)
N1	4 (8.00)	2 (9.52)
N2	3 (6.00)	9 (42.86)
N3	3 (6.00)	7 (33.33)
Clinical M stage, n (%)*		
M0	38 (72.00)	21 (100.00)
M1	3 (6.00)	0 (0.00)
Histological grade, n (%)**		
I	10 (20.00)	0 (0.00)
II	12 (24.00)	3 (14.29)
III	8 (16.00)	18 (85.71)

CIS, carcinoma *in situ*; IC, invasive carcinoma. *Followed the AJCC 8th edition staging guideline. **Followed the modified Scarff-Bloom-Richardson grade.

**Table 2:** Label-free MPM identifiable features of benign breast lesions, CIS, and IC.

Sample	Cell	Extracellular matrix	Texture
Benign	Two layers of epithelial cells line inside the duct	Curly and plenty of collagen fibers and elastin fibers proliferate in the stroma	The grey levels are evenly distributed in both TPEF and SHG signals
	The epithelial cells are regular, uniform, small-scale, and sparsely distributed	The diameter of the collagen fiber is large and uniform	
	The nuclei of the cells are small and rounded	Basement membrane of the duct is visible and complete	
CIS	Tumor cells proliferate in the duct	Linear and rich collagen fibers proliferate in the stroma	Increased heterogeneities in grey levels are shown in both TPEF and SHG signals
	Tumor cells with mild cytologic atypia are evenly spaced apart, and densely distributed	The diameter of the collagen fiber is small	
	The nuclei of the cells are medium-size, rounded or oval	Basement membrane of the duct is visible and complete, but significantly enlarged	
IC	Tumor cells proliferate and infiltrate into the stroma	Linear and sparse collagen fibers and fractured elastic fibers proliferate in the stroma	High heterogeneities in grey levels and abrupt changes in pixel intensities in both TPEF and SHG signals
	Tumor cells are large, irregular, and in high cytologic atypia	Collagen fibers vary greatly in diameters	
	The nuclei of the cells are large and irregular in shape	Basement membrane is disappeared	

**Table 3:** The performance metrics in different diagnostic models.

Models	Class	Accuracy (±SE)	Precision (±SE)	Recall (±SE)	F1-score (±SE)	Specificity (±SE)
DT22	Benign	62.79% (±5.82%)	51.43% (±11.59%)	67.86% (±14.14%)	55.24% (±10.26%)	80.60% (±6.21%)
	CIS		45.24% (±15.73%)	47.62% (±16.03%)	40.00% (±11.71%)	82.18% (±8.10%)
	IC		77.38% (±8.47%)	71.90% (±8.38%)	72.96% (±7.40%)	80.31% (±6.60%)
DT3	Benign	59.20% (±2.50%)	56.19% (±13.94%)	67.86% (±14.14%)	54.29% (±9.89%)	81.19% (±6.85%)
	CIS		40.48% (±16.10%)	42.86% (±15.79%)	30.48% (±8.18%)	78.71% (±7.73%)
	IC		76.43% (±8.58%)	67.14% (±5.93%)	69.69% (±5.39%)	80.31% (±6.60%)
MLP22	Benign	**93.65% (±4.10%)**	**92.86% (±7.14%)**	**100.00% (±0.00%)**	**95.24% (±4.76%)**	**98.21% (±1.79%)**
	CIS		**100.00% (±0.00%)**	**80.95% (±12.30%)**	**85.71% (±9.22%)**	**100.00% (±0.00%)**
	IC		**90.48% (±7.14%)**	**100.00% (±0.00%)**	**93.94% (±4.72%)**	**92.35% (±4.96%)**
MLP3	Benign	87.30% (±9.52%)	90.48% (±9.52%)	**100.00% (±0.00%)**	92.86% (±7.14%)	96.43% (±3.57%)
	CIS		85.71% (±14.29%)	76.19% (±15.79%)	7.86% (±14.87%)	92.86% (±7.14%)
	IC		88.10% (±7.90%)	91.43% (±8.57%)	88.10% (±7.90%)	92.35% (±4.96%)
RF22	Benign	70.04% (±5.70%)	68.57% (±14.21%)	71.43% (±13.83%)	65.76% (±11.61%)	93.45% (±3.13%)
	CIS		33.33% (±17.82%)	33.33% (±17.82%)	28.57% (±14.87%)	91.50% (±4.34%)
	IC		70.68% (±10.08%)	90.00% (±7.24%)	77.57% (±8.67%)	67.04% (±7.72%)
RF3	Benign	68.18% (±5.06%)	82.86% (±8.92%)	85.71% (±7.43%)	80.04% (±5.07%)	93.45% (±3.13%)
	CIS		28.57% (±18.44%)	9.52% (±6.15%)	14.29% (±9.22%)	91.84% (±6.12%)
	IC		69.29% (±6.68%)	91.90% (±5.81%)	76.76% (±5.40%)	61.46% (±5.79%)
SVM22	Benign	69.22% (±5.30%)	80.95% (±14.29%)	85.71% (±14.29%)	82.86% (±14.09%)	95.92% (±4.08%)
	CIS		34.29% (±17.84%)	28.57% (±13.47%)	30.00% (±14.64%)	82.31% (±7.27%)
	IC		68.57% (±7.52%)	71.43% (±8.96%)	68.21% (±7.58%)	69.93% (±4.81%)
SVM3	Benign	67.18% (±4.59%)	75.00% (±14.43%)	82.14% (±14.14%)	77.38% (±13.93%)	95.58% (±2.87%)
	CIS		34.29% (±17.84%)	23.81% (±11.98%)	25.71% (±12.70%)	82.31% (±7.27%)
	IC		67.35% (±7.31%)	73.81% (±9.07%)	68.81% (±7.66%)	67.07% (±4.66%)

Mean value ± standard error are reported. Best performed mean values are highlighted in bold face. SE, standard error.

## Abbreviations

MPM: Multiphoton Microscopy
H&E: Hematoxylin-eosin
MT: Masson's trichrome
IHC: Immunohistochemistry
pCR: Pathological Complete Response
MRI: Magnetic Resonance Imaging
OCT: Optical Coherence Tomography
LSCM: Laser Scanning Confocal Microscopy
TPEF: Two-photon Excitation Fluorescence
SHG: Second-harmonic Generation
FFPE: Formalin-fixed Paraffin-embedded
ROI: Regions of Interest
FFT: Fast Fourier Transform
CIS: Carcinoma *in situ*
IC: Invasive Carcinoma
TNBC: Triple-negative Breast Cancer
DT: Decision Tree
SVM: Support Vector Machine
RF: Random Forest
MLP: Multi-Layer Perceptron
NAIT: Neoadjuvant Immunotherapy
MINT: Multiphoton Imaging-clinical iNformation Two-stage machine learning model
AUROC: Area Under Receiver Operating Characteristic Curve
AUPRC: Area Under Precision-recall Curve

## References

[1] Elston CW, Ellis IO (1991). Pathological prognostic factors in breast cancer. I. The value of histological grade in breast cancer: experience from a large study with long-term follow-up. Histopathology.

[2] Bloom HJ, Richardson WW (1957). Histological grading and prognosis in breast cancer; a study of 1409 cases of which 359 have been followed for 15 years. Br J Cancer.

[3] Tan PH, Ellis I, Allison K, Brogi E, Fox SB, Lakhani S (2020). The 2019 World Health Organization classification of tumours of the breast. Histopathology.

[4] WHO Classification of Tumours Editorial Board (2019). Classification of Breast Tumours, 5th edn. Lyon: IARC Press.

[5] See SHC, Siziopikou KP (2022). Pathologic evaluation of specimens after neoadjuvant chemotherapy in breast cancer: Current recommendations and challenges. Pathol Res Pract.

[6] Jackman RJ, Marzoni FA Jr, Rosenberg J (2009). False-negative diagnoses at stereotactic vacuum-assisted needle breast biopsy: long-term follow-up of 1,280 lesions and review of the literature. AJR Am J Roentgenol.

[7] Shah VI, Raju U, Chitale D, Deshpande V, Gregory N, Strand V (2003). False-negative core needle biopsies of the breast: an analysis of clinical, radiologic, and pathologic findings in 27 concecutive cases of missed breast cancer. Cancer.

[8] Pillar N, Ozcan A (2022). Virtual tissue staining in pathology using machine learning. Expert Rev Mol Diagn.

[9] Ren W, Guo W, Kang D, Han Z, He J, Xi G (2021). Visualization of lymphatic vascular invasion in breast cancer by multiphoton microscopy. Lasers Med Sci.

[10] Tromberg BJ, Cerussi A, Shah N, Compton M, Durkin A, Hsiang D (2005). Imaging in breast cancer: diffuse optics in breast cancer: detecting tumors in pre-menopausal women and monitoring neoadjuvant chemotherapy. Breast Cancer Res.

[11] Kerlikowske K, Grady D, Barclay J, Sickles EA, Ernster V (1996). Likelihood ratios for modern screening mammography. Risk of breast cancer based on age and mammographic interpretation. JAMA.

[12] Hindle WH, Davis L, Wright D (1999). Clinical value of mammography for symptomatic women 35 years of age and younger. Am J Obstet Gynecol.

[13] Kopans DB (1999). Breast-cancer screening with ultrasonography. Lancet.

[14] Buchberger W, Niehoff A, Obrist P, DeKoekkoek-Doll P, Dunser M (2000). Clinically and mammographically occult breast lesions: detection and classification with high-resolution sonography. Semin Ultrasound CT MR.

[15] Shin HJ, Kim HH, Cha JH, Park JH, Lee KE, Kim JH (2011). Automated ultrasound of the breast for diagnosis: interobserver agreement on lesion detection and characterization. AJR Am J Roentgenol.

[16] Han Z, Li L, Kang D, Zhan Z, Tu H, Wang C (2019). Label-free detection of residual breast cancer after neoadjuvant chemotherapy using biomedical multiphoton microscopy. Lasers Med Sci.

[17] Bombardieri E, Gianni L (2004). The choice of the correct imaging modality in breast cancer management. Eur J Nucl Med Mol Imaging.

[18] Hoover EE, Squier JA (2013). Advances in multiphoton microscopy technology. Nat Photonics.

[19] Tilbury K, Campagnola PJ (2015). Applications of second-harmonic generation imaging microscopy in ovarian and breast cancer. Perspect Medicin Chem.

[20] Alexander S, Weigelin B, Winkler F, Friedl P (2013). Preclinical intravital microscopy of the tumour-stroma interface: invasion, metastasis, and therapy response. Curr Opin Cell Biol.

[21] Jain M, Narula N, Aggarwal A, Stiles B, Shevchuk MM, Sterling J (2014). Multiphoton microscopy: a potential "optical biopsy" tool for real-time evaluation of lung tumors without the need for exogenous contrast agents. Arch Pathol Lab Med.

[22] Campagnola PJ, Loew LM (2003). Second-harmonic imaging microscopy for visualizing biomolecular arrays in cells, tissues and organisms. Nat Biotechnol.

[23] Brown E, McKee T, diTomaso E, Pluen A, Seed B, Boucher Y (2003). Dynamic imaging of collagen and its modulation in tumors *in vivo* using second-harmonic generation. Nat Med.

[24] Dudenkova VV, Shirmanova MV, Lukina MM, Feldshtein FI, Virkin A, Zagainova EV (2019). Examination of Collagen Structure and State by the Second Harmonic Generation Microscopy. Biochemistry (Mosc).

[25] Fang M, Yuan J, Peng C, Li Y (2014). Collagen as a double-edged sword in tumor progression. Tumour Biol.

[26] Wyckoff JB, Wang Y, Lin EY, Li JF, Goswami S, Stanley ER (2007). Direct visualization of macrophage-assisted tumor cell intravasation in mammary tumors. Cancer Res.

[27] Giese A, Kluwe L, Laube B, Meissner H, Berens ME, Westphal M (1996). Migration of human glioma cells on myelin. Neurosurgery.

[28] Wolfe JN (1976). Risk for breast cancer development determined by mammographic parenchymal pattern. Cancer.

[29] Wu X, Chen G, Qiu J, Lu J, Zhu W, Chen J (2016). Visualization of basement membranes in normal breast and breast cancer tissues using multiphoton microscopy. Oncol Lett.

[30] Wu X, Chen G, Lu J, Zhu W, Qiu J, Chen J (2013). Label-free detection of breast masses using multiphoton microscopy. PLoS One.

[31] Wu Y, Lin Y, Lian Y, Lin P, Wang S, Fu F (2018). Identifying Two Common Types of Breast Benign Diseases Based on Multiphoton Microscopy. Scanning.

[32] Chen Z, Guo W, Kang D, Wang S, Zheng L, Xi G (2020). Label-Free Identification of Early Stages of Breast Ductal Carcinoma via Multiphoton Microscopy. Scanning.

[33] Matsui T, Iwasa A, Mimura M, Taniguchi S, Sudo T, Uchida Y (2022). Label-free multiphoton excitation imaging as a promising diagnostic tool for breast cancer. Cancer Sci.

[34] Wu Y, Fu F, Lian Y, Chen J, Wang C, Nie Y (2015). Monitoring morphological alterations during invasive ductal breast carcinoma progression using multiphoton microscopy. Lasers Med Sci.

[35] Xi G, He J, Kang D, Xu S, Guo W, Fu F (2021). Nomogram model combining macro and micro tumor-associated collagen signatures obtained from multiphoton images to predict the histologic grade in breast cancer. Biomed Opt Express.

[36] Desa DE, Wu W, Brown RM, Brown EBt, Hill RL, Turner BM (2022). Second-Harmonic Generation Imaging Reveals Changes in Breast Tumor Collagen Induced by Neoadjuvant Chemotherapy. Cancers (Basel).

[37] Li L, Han Z, Qiu L, Kang D, Zhan Z, Tu H (2020). Label-free multiphoton imaging to assess neoadjuvant therapy responses in breast carcinoma. Int J Biol Sci.

[38] Han Z, Huang X, Kang D, Fu F, Zhang S, Zhan Z (2023). Detection of pathological response of axillary lymph node metastasis after neoadjuvant chemotherapy in breast cancer using multiphoton microscopy. J Biophotonics.

[39] Desa DE, Strawderman RL, Wu W, Hill RL, Smid M, Martens JWM (2020). Intratumoral heterogeneity of second-harmonic generation scattering from tumor collagen and its effects on metastatic risk prediction. BMC Cancer.

[40] Gole L, Yeong J, Lim JCT, Ong KH, Han H, Thike AA (2020). Quantitative stain-free imaging and digital profiling of collagen structure reveal diverse survival of triple negative breast cancer patients. Breast Cancer Res.

[41] He J, Kang D, Xu M, Han Z, Guo W, Fu F (2023). Combining the guidelines and multiphoton imaging methods to improve the prognostic value of tumor-infiltrating lymphocytes in breast cancer. J Biophotonics.

[42] Chen J, Li Z, Han Z, Kang D, Ma J, Yi Y (2023). Prognostic value of tumor necrosis based on the evaluation of frequency in invasive breast cancer. BMC Cancer.

[43] He J, Fu F, Wang W, Xi G, Guo W, Zheng L (2021). Prognostic value of tumour-infiltrating lymphocytes based on the evaluation of frequency in patients with oestrogen receptor-positive breast cancer. Eur J Cancer.

[44] Case A, Brisson BK, Durham AC, Rosen S, Monslow J, Buza E (2017). Identification of prognostic collagen signatures and potential therapeutic stromal targets in canine mammary gland carcinoma. PLoS One.

[45] Huang X, Fu F, Guo W, Kang D, Han X, Zheng L (2024). Prognostic significance of collagen signatures at breast tumor boundary obtained by combining multiphoton imaging and imaging analysis. Cell Oncol (Dordr).

[46] Ghazaryan A, Tsai HF, Hayrapetyan G, Chen WL, Chen YF, Jeong MY (2013). Analysis of collagen fiber domain organization by Fourier second harmonic generation microscopy. J Biomed Opt.

[47] Matteini P, Ratto F, Rossi F, Cicchi R, Stringari C, Kapsokalyvas D (2009). Photothermally-induced disordered patterns of corneal collagen revealed by SHG imaging. Opt Express.

[48] Rao RA, Mehta MR, Toussaint KC Jr (2009). Fourier transform-second-harmonic generation imaging of biological tissues. Opt Express.

[49] Wu S, Li H, Yang H, Zhang X, Li Z, Xu S (2011). Quantitative analysis on collagen morphology in aging skin based on multiphoton microscopy. J Biomed Opt.

[50] Hotaling NA, Bharti K, Kriel H, Simon CG Jr (2015). DiameterJ: A validated open source nanofiber diameter measurement tool. Biomaterials.

[51] Uckermann O, Galli R, Mark G, Meinhardt M, Koch E, Schackert G (2020). Label-free multiphoton imaging allows brain tumor recognition based on texture analysis-a study of 382 tumor patients. Neurooncol Adv.

[52] Raub CB, Putnam AJ, Tromberg BJ, George SC (2010). Predicting bulk mechanical properties of cellularized collagen gels using multiphoton microscopy. Acta Biomater.

[53] Wang XQ, Danenberg E, Huang CS, Egle D, Callari M, Bermejo B (2023). Spatial predictors of immunotherapy response in triple-negative breast cancer. Nature.

[54] Schmid P, Cortes J, Dent R, Pusztai L, McArthur H, Kummel S (2022). Event-free Survival with Pembrolizumab in Early Triple-Negative Breast Cancer. N Engl J Med.

[55] Desa DE, Bhanote M, Hill RL, Majeski JB, Buscaglia B, D'Aguiar M (2019). Second-harmonic generation directionality is associated with neoadjuvant chemotherapy response in breast cancer core needle biopsies. J Biomed Opt.

[56] Brown Y, Hua S, Tanwar PS (2019). Extracellular matrix-mediated regulation of cancer stem cells and chemoresistance. Int J Biochem Cell Biol.

[57] Senthebane DA, Rowe A, Thomford NE, Shipanga H, Munro D, Mazeedi M (2017). The Role of Tumor Microenvironment in Chemoresistance: To Survive, Keep Your Enemies Closer. Int J Mol Sci.

[58] Correia AL, Bissell MJ (2012). The tumor microenvironment is a dominant force in multidrug resistance. Drug Resist Updat.

[59] Hanahan D, Weinberg RA (2011). Hallmarks of cancer: the next generation. Cell.

[60] Hanahan D (2022). Hallmarks of Cancer: New Dimensions. Cancer Discov.

[61] Sherr CJ, McCormick F (2002). The RB and p53 pathways in cancer. Cancer Cell.

[62] Polyak K, Haviv I, Campbell IG (2009). Co-evolution of tumor cells and their microenvironment. Trends Genet.

[63] Vander Heiden MG, Cantley LC, Thompson CB (2009). Understanding the Warburg effect: the metabolic requirements of cell proliferation. Science.

[64] Kessenbrock K, Plaks V, Werb Z (2010). Matrix metalloproteinases: regulators of the tumor microenvironment. Cell.

[65] Egeblad M, Nakasone ES, Werb Z (2010). Tumors as organs: complex tissues that interface with the entire organism. Dev Cell.

[66] Qian BZ, Pollard JW (2010). Macrophage diversity enhances tumor progression and metastasis. Cell.

[67] Kai F, Drain AP, Weaver VM (2019). The Extracellular Matrix Modulates the Metastatic Journey. Dev Cell.

[68] Maller O, Drain AP, Barrett AS, Borgquist S, Ruffell B, Zakharevich I (2021). Tumour-associated macrophages drive stromal cell-dependent collagen crosslinking and stiffening to promote breast cancer aggression. Nat Mater.

[69] Fiore VF, Krajnc M, Quiroz FG, Levorse J, Pasolli HA, Shvartsman SY (2020). Mechanics of a multilayer epithelium instruct tumour architecture and function. Nature.

[70] Panciera T, Citron A, Di Biagio D, Battilana G, Gandin A, Giulitti S (2020). Reprogramming normal cells into tumour precursors requires ECM stiffness and oncogene-mediated changes of cell mechanical properties. Nat Mater.

[71] Li L, Han Z, Qiu L, Kang D, Zhan Z, Tu H (2020). Evaluation of breast carcinoma regression after preoperative chemotherapy by label-free multiphoton imaging and image analysis. J Biophotonics.

[72] Naik A, Leask A (2023). Tumor-associated fibrosis impairs the response to immunotherapy. Matrix Biol.

[73] Xiao Z, Todd L, Huang L, Noguera-Ortega E, Lu Z, Huang L (2023). Desmoplastic stroma restricts T cell extravasation and mediates immune exclusion and immunosuppression in solid tumors. Nat Commun.

[74] Egeblad M, Rasch MG, Weaver VM (2010). Dynamic interplay between the collagen scaffold and tumor evolution. Curr Opin Cell Biol.

[75] Provenzano PP, Eliceiri KW, Campbell JM, Inman DR, White JG, Keely PJ (2006). Collagen reorganization at the tumor-stromal interface facilitates local invasion. BMC Med.

[76] Sleeboom JJF, van Tienderen GS, Schenke-Layland K, van der Laan LJW, Khalil AA, Verstegen MMA (2024). The extracellular matrix as hallmark of cancer and metastasis: From biomechanics to therapeutic targets. Sci Transl Med.

[77] Sabeh F, Shimizu-Hirota R, Weiss SJ (2009). Protease-dependent versus -independent cancer cell invasion programs: three-dimensional amoeboid movement revisited. J Cell Biol.

[78] Baba Y, Iyama K, Ikeda K, Ishikawa S, Hayashi N, Miyanari N (2008). The expression of type IV collagen alpha6 chain is related to the prognosis in patients with esophageal squamous cell carcinoma. Ann Surg Oncol.

[79] Cambria E, Coughlin MF, Floryan MA, Offeddu GS, Shelton SE, Kamm RD (2024). Linking cell mechanical memory and cancer metastasis. Nat Rev Cancer.

[80] Piersma B, Hayward MK, Weaver VM (2020). Fibrosis and cancer: A strained relationship. Biochim Biophys Acta Rev Cancer.

[81] Wu T, Xiong S, Chen M, Tam BT, Chen W, Dong K (2023). Matrix stiffening facilitates the collective invasion of breast cancer through the periostin-integrin mechanotransduction pathway. Matrix Biol.

[82] Fitzgerald KA, Guo J, Tierney EG, Curtin CM, Malhotra M, Darcy R (2015). The use of collagen-based scaffolds to simulate prostate cancer bone metastases with potential for evaluating delivery of nanoparticulate gene therapeutics. Biomaterials.

[83] Wu Y, Fu F, Lian Y, Nie Y, Zhuo S, Wang C (2015). Monitoring the progression from intraductal carcinoma to invasive ductal carcinoma based on multiphoton microscopy. J Biomed Opt.

