# Supplementary material for: Quantitative Histopathology Analysis Based on Label-free Multiphoton Imaging for Breast Cancer Diagnosis and Neoadjuvant Immunotherapy Response Assessment
Source: Int J Biol Sci. 2025 Jan 1;21(1):363–81. doi: 10.7150/ijbs.102744 (PMC11667811; doi:10.7150/ijbs.102744)
Supplement: Supplementary file 1 — Supplementary figures and tables. [file ijbsv21p0363s1.pdf]

Supplementary Table 1. Characteristics of BC patients in CJFH cohort.

Number	Histological classification	Age	Molecular subtype	Tumor size	Nodal status	Metastatic status	cTNM (cStage)*	Histological grade**	Tissue type
1	Benign	26	NA	NA	-	-	NA	NA	adenosis tissue
2	CIS	66	Luminal A	0.8	-	-	TisN0M0 (0)	NA	both normal and tumor tissues
3	CIS	50	Luminal A	1.1	-	-	TisN0M0 (0)	NA	both normal and tumor tissues
4	CIS	67	Luminal B	1.5	-	-	TisN0M0 (0)	NA	both normal and tumor tissues
5	CIS	69	Luminal B	1.4	-	-	TisN0M0 (0)	NA	tumor tissue
6	CIS	53	Luminal B	3.5	-	-	TisN0M0 (0)	NA	tumor tissue
7	CIS	40	HER2	3	-	-	TisN0M0 (0)	NA	both normal and tumor tissues
8	CIS	65	Luminal B	1.2	-	-	TisN0M0 (0)	NA	both normal and tumor tissues
9	CIS	39	Luminal A	3	-	-	TisN0M0 (0)	NA	tumor tissue
10	CIS	54	HER2	3.5	-	-	TisN0M0 (0)	NA	tumor tissue
11	CIS	45	Luminal B	6	-	-	TisN0M0 (0)	NA	tumor tissue
12	IC	48	Luminal A	1.5	-	-	T1N0M0 (I)	II	both normal and tumor tissues
13	IC	71	TNBC	2.8	-	-	T2N0M0 (II)	III	tumor tissue
14	IC	36	Luminal A	1.2	+	-	T1N1M0 (II)	II	tumor tissue
15	IC	61	Luminal B	2	+	-	T1N2M0 (III)	III	tumor tissue
16	CIS	40	Luminal B	3	-	-	TisN0M0 (0)	NA	tumor tissue
17	CIS	35	TNBC	0.4	-	-	TisN0M0 (0)	NA	tumor tissue

18	IC	62	Luminal B	1.5	-	-	T1N0M0 (I)	II	tumor tissue
19	IC	60	Luminal B	4	-	+	T2N0M1 (IV)	III	tumor tissue
20	IC	77	TNBC	5	-	-	T2N0M0 (II)	III	tumor tissue
21	IC	45	Luminal B	2.8	+	-	T2N3M0 (III)	II	tumor tissue
22	IC	82	Luminal B	2.8	-	-	T2N0M0 (II)	II	tumor tissue
23	IC	61	NA	3.5	-	-	T2N0M0 (II)	II	tumor tissue
24	IC	58	Luminal B	2.5	+	-	T2N3M0 (III)	I	both normal and tumor tissues
25	IC	64	Luminal B	1.2	-	-	T1N0M0 (I)	II	both normal and tumor tissues
26	IC	66	Luminal A	3	+	+	T2N3M1 (IV)	II	tumor tissue
27	Benign	44	NA	NA	-	-	NA	NA	adenosis tissue
28	IC	76	Luminal B	1.5	-	-	T1N0M0 (I)	II	tumor tissue
29	IC	50	Luminal A	2.8	-	-	T2N0M0 (II)	II	tumor tissue
30	IC	51	Luminal A	NA	NA	NA	NA	II	tumor tissue
31	IC	65	Luminal B	2.5	-	-	T2N0M0 (II)	II	tumor tissue
32	IC	52	Luminal B	1.5	-	-	T1N0M0 (I)	I	tumor tissue
33	IC	66	Luminal B	2	-	-	T1N0M0 (I)	I	tumor tissue
34	IC	43	Luminal A	1.3	+	-	T1N1M0 (II)	I	tumor tissue
35	Benign	32	NA	NA	-	-	NA	NA	adenosis tissue
36	Benign	63	NA	NA	-	-	NA	NA	adenosis tissue
37	Benign	41	NA	NA	-	-	NA	NA	adenosis tissue
38	CIS	72	Luminal A	3.7	-	-	TisN0M0 (0)	NA	tumor tissue
39	CIS	56	Luminal B	NA	-	-	TisN0M0 (0)	NA	both normal and tumor tissues

40	CIS	71	Luminal B	0.3	-	-	TisN0M0 (0)	NA	tumor tissue
41	IC	46	Luminal B	1.6	-	-	T1N0M0 (I)	I	tumor tissue
42	IC	80	Luminal B	2.7	NA	NA	NA	III	tumor tissue
43	IC	45	Luminal A	1	-	-	T1N0M0 (I)	I	both normal and tumor tissues
44	IC	73	TNBC	NA	NA	NA	NA	III	tumor tissue
45	IC	40	Luminal B	1.2	+	-	T1N1M0 (II)	I	tumor tissue
46	IC	75	Luminal B	2	+	-	T1N1M0 (II)	I	both normal and tumor tissues
47	IC	42	Luminal A	1.8	+	-	T1N2M0 (III)	I	tumor tissue
48	IC	74	Luminal B	2.5	-	-	T2N0M0 (II)	III	tumor tissue
49	IC	44	HER2	NA	NA	NA	NA	III	tumor tissue
50	IC	72	Luminal A	5.5	+	+	T4N2M1 (IV)	I	both normal and tumor tissues

NA, not applicable; DCIS, ductal carcinoma in situ; IDC, invasive ductal carcinoma; ILC, invasive lobular carcinoma; TNBC, triple-negative breast cancer.

*Followed the AJCC 8th edition staging guideline.

**Followed the modified Scarff-Bloom-Richardson grade.

Supplementary Table 2. Characteristics of BC patients treated with immune-neoadjuvant therapy in PUCH cohort.

Number	Histological classification before treatment	Age	Molecular subtype	Tumor size	Nodal status	cTNM (cStage)*	Histological grade	Tissue type	Miller-Payne grade**	pCR	Therapeutic evaluation***
1	IC	39	TNBC	4.8	+	T2N1M0 (II)	III	tumor tissues before & afte	2	N	PR
2	IC	55	TNBC	2.3	+	T2N3M0 (III)	II		5	N	PR
3	IC	63	TNBC	5.2	+	T4N3M0 (III)	II		1	N	SD

4	IC	55	TNBC	4	+	T2N2 M0 (III)	III	r treatment	4	N	PR
5	IC	53	TNBC	2.5	+	T2N3 M0 (III)	III		5	Y	PR
6	IC	46	TNBC	1.7	+	T1N2 M0 (III)	III		3	N	SD
7	IC	34	TNBC	2	+	T2N2 M0 (III)	III		2	N	SD
8	IC	52	TNBC	3.3	+	T2N2 M0 (III)	III		3	N	PR
9	IC	56	TNBC	2.5	+	T2N1 M0 (II)	III		3	N	PR
10	IC	41	TNBC	4.4	+	T3N3 M0 (III)	III		2	N	PR
11	IC	46	TNBC	3.6	+	T2N2 M0 (III)	III		4	N	PR
12	IC	60	TNBC	2.5	+	T2N3 M0 (III)	II		3	N	SD
13	IC	54	TNBC	3	-	T2N0 M0 (II)	III		5	Y	PR
14	IC	63	TNBC	2.6	-	T2N0 M0 (II)	III		5	Y	CR
15	IC	56	TNBC	2.8	+	T2N2 M0 (III)	III		5	Y	SD
16	IC	37	TNBC	2.5	+	T2N2 M0 (III)	III		5	Y	PR
17	IC	70	TNBC	2.3	+	T2N3 M0 (III)	III		5	Y	PR

18	IC	45	TNBC	3.4	+	T2N2 M0 (III)	III	5	Y	PR
19	IC	45	TNBC	3.0	+	T2N2 M0 (III)	III	5	Y	PR
20	IC	33	TNBC	3.2	+	T2N0 M0 (II)	III	5	Y	PR
21	IC	63	TNBC	5	+	T3N3 M0 (III)	III	5	N	PR

IC, invasive carcinoma; TNBC, triple-negative breast cancer; pCR, pathologic complete response; N, no; Y, yes; CR, complete response; PR, partial response, SD, stable disease; PD, progressive disease.

*Followed the AJCC 8th edition staging guideline.

**Followed the modified Scarff-Bloom-Richardson grade.

***Followed the RECIST-1.1 guideline.

Supplementary Table 3. List of 24 features constructed from label-free MPM images.

	Feature name	Explanation	Method and formula
1	Cell: mean of cell area	The average area of ductal epithelial cell for one slice	Manually tracing the outer boundary of the selected cell (with TPEF signal) and subsequently calculating its area, followed by determining the average area across all 20 cells.
2	Cell: stdev of cell area	The standard deviation of ductal epithelial cell for one slice	Calculate the cell area as mentioned above, followed by determining the standard deviation across all 20 cells.
3	Cell: mean of nucleus area	The average nucleus area of ductal epithelial cell for one slice	Manually tracing the outer boundary of the nucleus of the selected cell (the dark oval structure located within the cell that does not exhibit a TPEF signal) and subsequently calculating its area, followed by determining the average area across all 20 cells.
4	Cell: stdev of nucleus area	The standard deviation of nucleus ductal epithelial cell for one slice	Calculate the nucleus as mentioned above, followed by determining the standard deviation across all 20 cells.
5	Cell: mean of nucleus-cytoplasm ratio	The average ratio of nucleus-cytoplasm for one slice	Manually tracing the outer boundary of the nucleus and the selected cell as mentioned above and subsequently calculating their areas, using nucleus area/ (cell area - nucleus area) to calculate the nucleus-cytoplasm ratio

			for each cell, followed by determining the average ratio across all 20 cells.
6	Cell: stdev of nucleus-cytoplasm ratio	The standard deviation of nucleus-cytoplasm ratio for one slice	Calculate nucleus-cytoplasm ratio for each cell as mentioned above, followed by determining the standard deviation across all 20 cells.
7	Extracellular matrix: mean of elastin density	The average area ratio of elastin to ROI for one slice	Open the TPEF channel image in ImageJ software, adjust the threshold to select the fibrous structures (elastin), calculate their area, and determine the elastin density within the ROI using the formula: elastin area / ROI area. Finally, calculate the average elastin density for 20 ROIs.
8	Extracellular matrix: stdev of elastin density	The standard deviation of area ratio of elastin to ROI for one slice	Calculate the elastin density for each ROI as mentioned above. Then, calculate the standard deviation of elastin density for 20 ROIs.
9	Extracellular matrix: mean of collagen density	The average area ratio of collagen to ROI for one slice	Open the SHG channel image in ImageJ software, adjust the threshold to select the fibrous structures (collagen), calculate their area, and determine the collagen density within the ROI using the formula: collagen area / ROI area. Finally, calculate the average collagen density for 20 ROIs.
10	Extracellular matrix: stdev of collagen density	The standard deviation of area ratio of collagen to ROI for one slice	Calculate the collagen density for each ROI as mentioned above. Then, calculate the standard deviation of collagen density for 20 ROIs.
11	Extracellular matrix: mean of elastin-collagen density ratio	The average area ratio of elastin to collagen for one slice	Calculate the elastin and collagen density of each ROI as mentioned above. Then determine the elastin-collagen density ratio using the formula: elastin density / collagen density. Finally, calculate the average ratio for 20 ROIs.
12	Extracellular matrix: stdev of elastin-collagen density ratio	The standard deviation of area ratio of elastin to collagen for one slice	Calculate the elastin-collagen density ratio for each ROI as mentioned above. Then, calculate the standard deviation of elastin-collagen density ratio for 20 ROIs.
13	Extracellular matrix: mean of	The average of 1-minor/major axis for	Perform the Fast Fourier Transform in

	collagen orientation	one slice	SHG channel images, and then used the ImageJ software for ellipse fitting and measured the long (L) and short (S) axes, characterized collagen fiber orientation by 1-S/L for each ROI. Finally, calculate the average value for 20 ROIs.
14	Extracellular matrix: stdev of collagen orientation	The standard deviation of 1-minor/major axis for one slice	Calculate 1-S/L for each ROI. Then, calculate the standard deviation of 1-S/L for 20 ROIs.
15	Extracellular matrix: mean of collagen diameter	The average diameter of collagen for one slice	Open the SHG channel image in ImageJ software and analyze the average diameter of collagen in each ROI with the DiameterJ plug-in in ImageJ. Finally, calculate the average diameter for 20 ROIs.
16	Extracellular matrix: stdev of collagen diameter	The standard deviation of average diameter for one slice	Calculate the average diameter of collagen in each ROI as mentioned above. Then, calculate the standard deviation of the average diameters of 20 ROIs.
17	Texture: mean of elastin intensity	The average integrated intensity of elastin for one slice	Open the TPEF channel image in ImageJ software, adjust the threshold to select the fibrous structures (elastin), calculate their intensity. Finally, calculate the average intensity of elastin for 20 ROIs.
18	Texture: stdev of elastin intensity	The standard deviation of integrated intensity of elastin for one slice	Calculate the intensity of elastin of each ROI as mentioned above. Then, calculate the standard deviation of the intensity for 20 ROIs.
19	Texture: mean of collagen intensity	The average integrated intensity of collagen for one slice	Open the SHG channel image in ImageJ software, adjust the threshold to select the fibrous structures (collagen), calculate their intensity. Finally, calculate the average intensity of collagen for 20 ROIs.
20	Texture: stdev of collagen intensity	The standard deviation of integrated intensity of collagen for one slice	Calculate the intensity of collagen of each ROI as mentioned above. Then, calculate the standard deviation of the intensity for 20 ROIs.
21	Texture: mean of average elastin intensity	The average ratio of elastin intensity to elastin density for one slice	Calculate the intensity of elastin and the elastin area for each ROI as

			mentioned above. Then, calculate the average elastin intensity by using the formula: intensity of elastin / elastin area. Finally, determine the mean of average elastin intensity for 20 ROIs.
22	Texture: stdev of average elastin intensity	The standard deviation of elastin intensity to elastin density ratio	Calculate the average elastin intensity for each ROI as mentioned above. Then, determine the standard deviation of average elastin intensity of 20 ROIs.
23	Texture: mean of average collagen intensity	The average ratio of collagen intensity to collagen density for one slice	Calculate the intensity of collagen and the collagen area for each ROI as mentioned above. Then, calculate the average collagen intensity by using the formula: intensity of collagen / collagen area. Finally, determine the mean of average collagen intensity for 20 ROIs.
24	Texture: stdev of average collagen intensity	The standard deviation of collagen intensity to collagen density ratio	Calculate the average collagen intensity for each ROI as mentioned above. Then, determine the standard deviation of average collagen intensity of 20 ROIs.

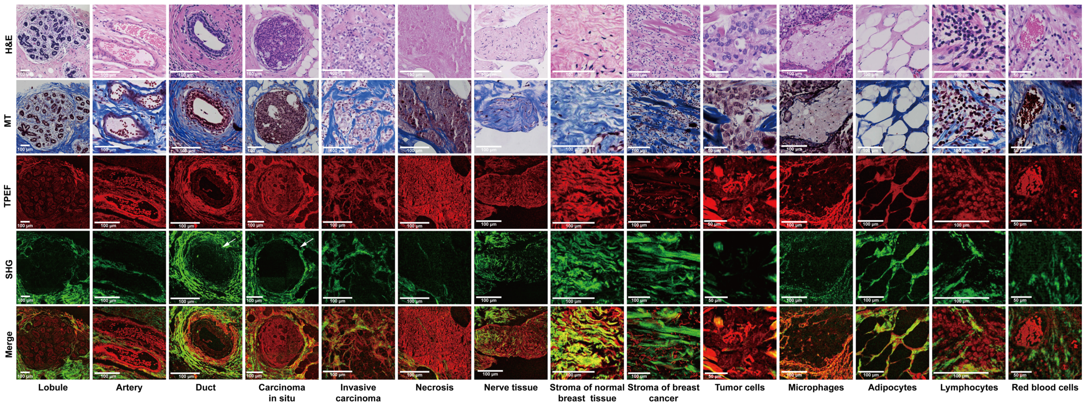

Supplementary Figure 1. The atlas of MPM images and corresponding H&E and Masson images of microstructure of tissues and cells in breast lesion tissues. The first and second lines display the H&E and MT-stained images of typical tissue structure and cells in beast lesions, respectively, while the corresponding label-free MPM images are presented below. In particular, the third line shows cells, and elastic fibers capable of producing TPEF signals (red color-coded), while the fourth line shows collagen fibers in the extracellular matrix producing SHG signals (green color-coded). White arrow: complete basement membrane. Scale bar, 100 μm.

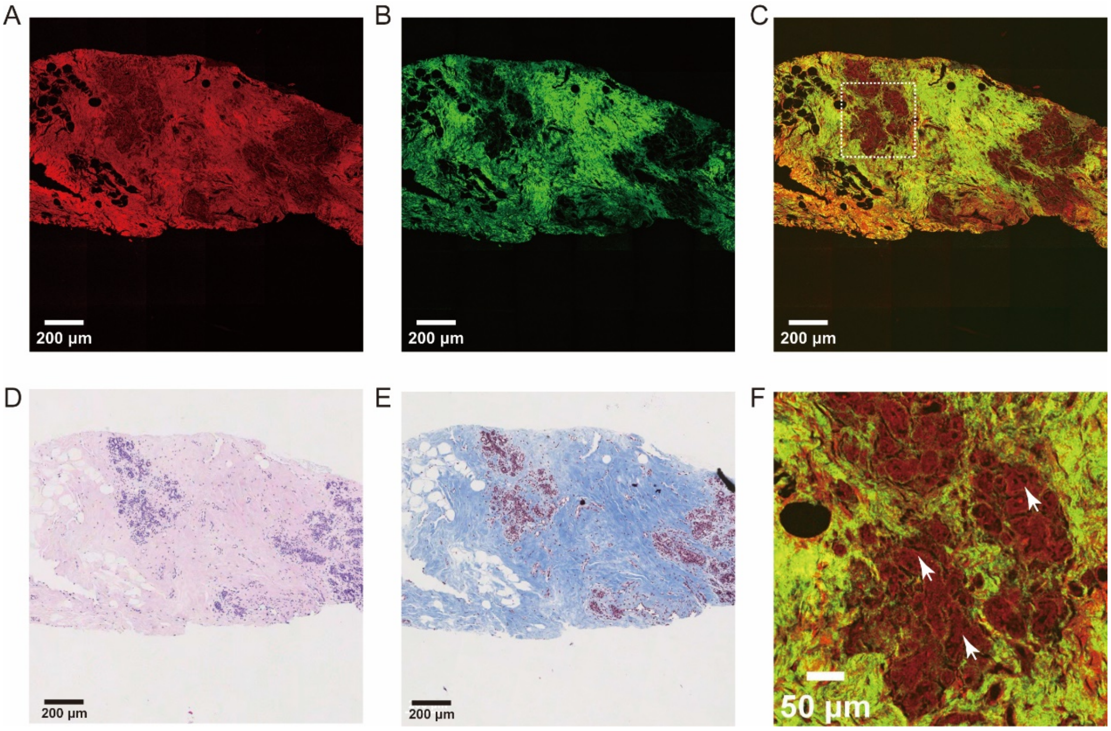

Supplementary Figure 2. Representative MPM images and corresponding H&E/Masson-stained image of benign breast lesion. **(A)** TPEF image; **(B)** SHG image; **(C)** Merged image; **(D)** H&E-stained image; **(E)** Masson-stained image; **(F)** Zoom-in overlaid image of the white dashed box region in (C). White arrow: acinus; scale bar: 200 μm (A-E) and 50 μm (F).

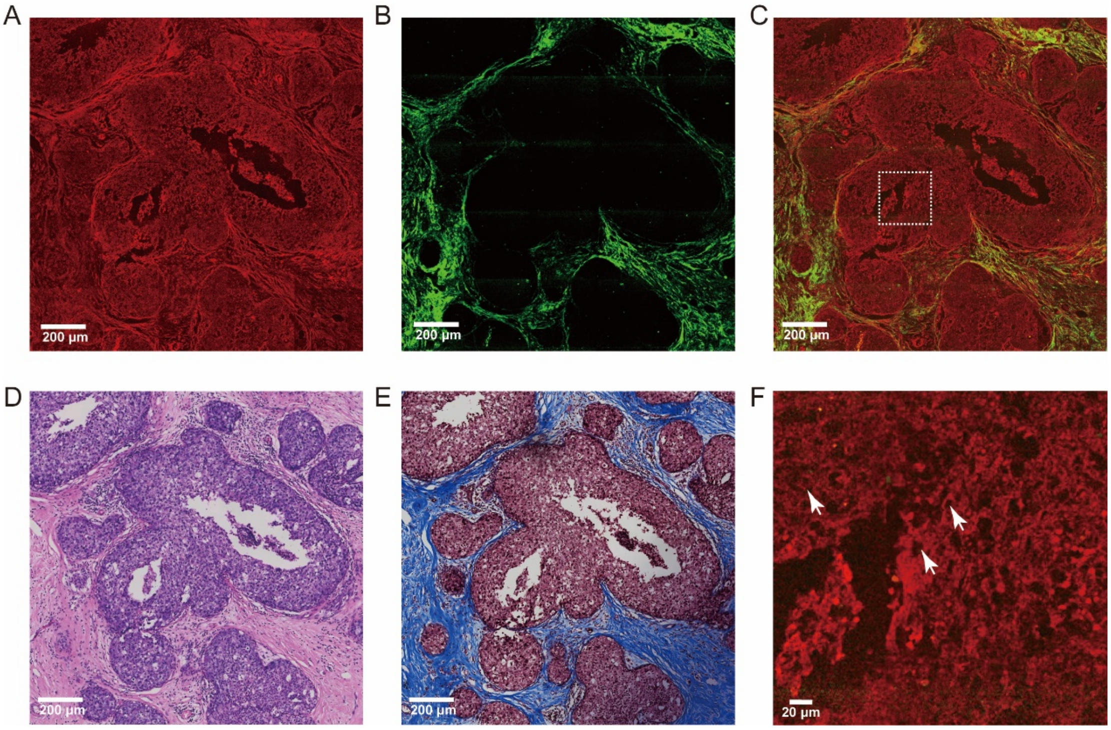

Supplementary Figure 3. Representative MPM images and corresponding H&E/Masson-stained image of CIS tissue. (A) TPEF image; **(B)** SHG image; **(C)** Merged image; **(D)** H&E-stained image; **(E)** Masson-stained image; **(F)** Zoom-in overlaid image of the white dashed box region in (C). White arrow: Tumor cell; scale bar: 200 μm (A-E) and 20 μm (F).

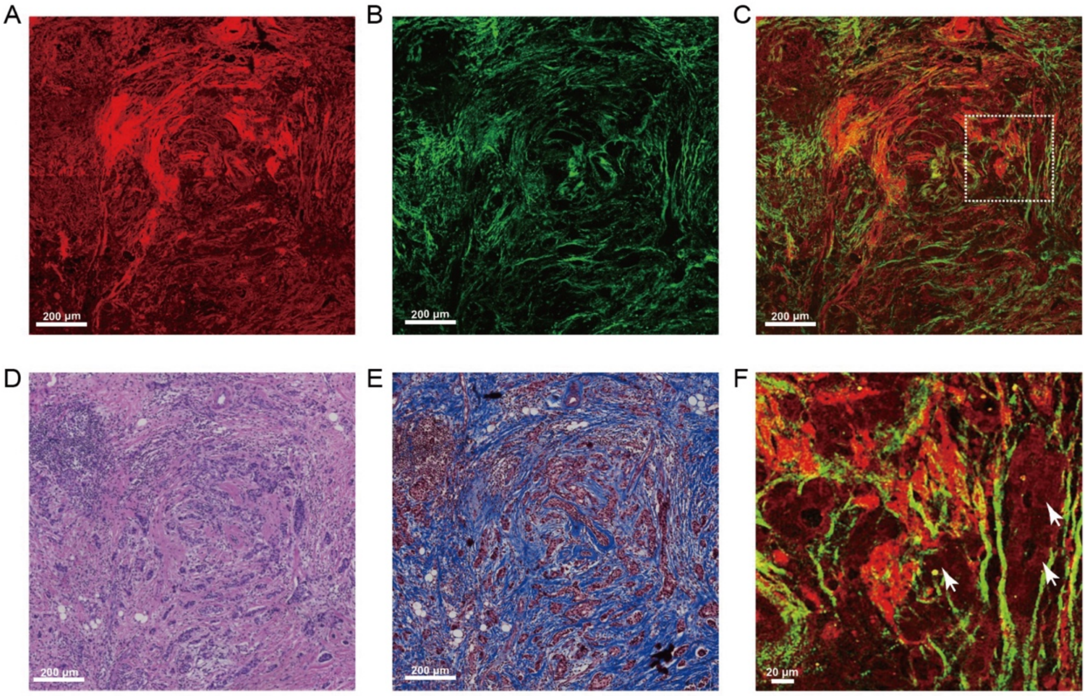

Supplementary Figure 4. Representative MPM images and corresponding H&E/Masson-stained image of IC tissue. (A) TPEF image; **(B)** SHG image; **(C)** Overlaid image; **(D)** H&E-

stained image; **(E)** Masson-stained image; **(F)** Zoom-in overlaid image of the white dashed box region in **(C)**. White arrow: Tumor cell; scale bar: 200 μm (A-E) and 20 μm (F).

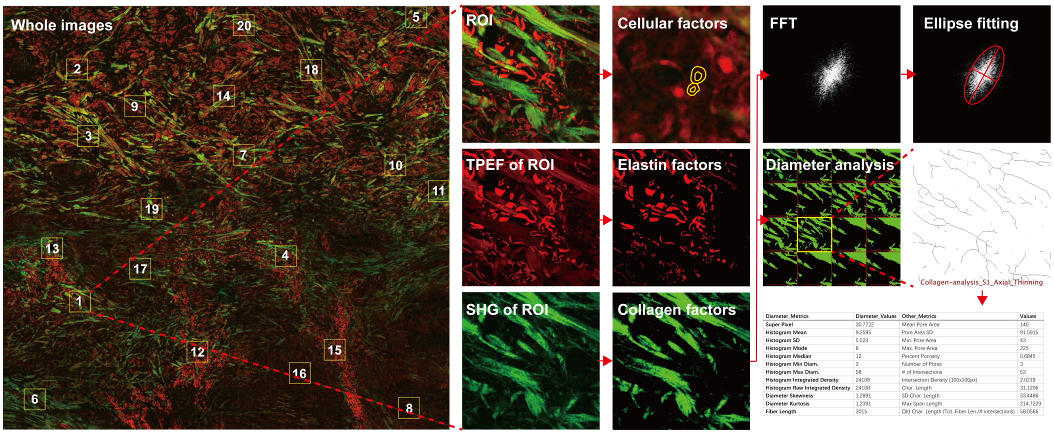

Supplementary Figure 5. The feature extraction pipeline. We randomly selected 20 ROIs (region of interest) of 512×512 pixels^2 on the suspicious lesion area of each label-free MPM image (shown on the left). Subsequently, we utilized merged images along with TPEF and SHG channel images to extract cell, elastic fibers, and collagen fiber features, respectively. As depicted in the middle, we manually outlined cells and nuclei to measure the mean and corresponding standard deviation of cell area, nucleus area, and nucleus-cytoplasm ratio. Elastic fibers were identified by adjusting thresholds in the TPEF channel, and we calculated the mean values and corresponding standard deviations of elastin density, elastin intensity, and average elastin intensity. The same approach was employed for collagen fibers in the SHG channel. Besides, the elastin-collagen density ratio was determined. Moreover, we evaluated the orientation of collagen fibers by employing a fast Fourier transform (FFT), followed by ellipse fitting and calculation of 1-S/L. Additionally, we utilized DiameterJ to measure the diameter of collagen fibers. Firstly, we conducted automatic segmentation of collagen fibers to determine the optimal segmentation approach; subsequently, DiameterJ was used to analyze the average diameter of collagen fibers (shown on the right).

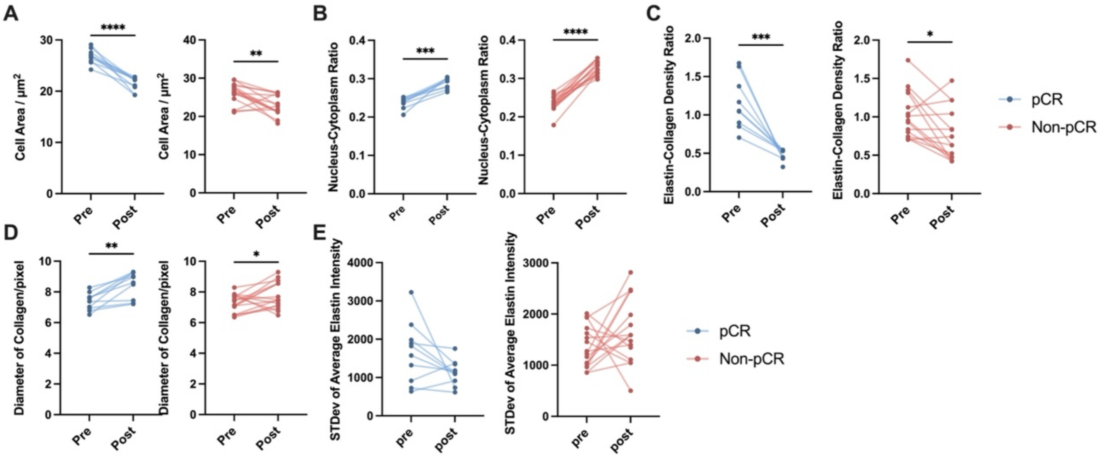

Supplementary Figure 6. Dynamic changes in key label-free MPM factors pre- and post-neoadjuvant immunotherapy in patients with breast cancer. (A) Mean of cell area; **(B)** mean of nucleus-cytoplasm ratio; **(C)** mean of elastin-collagen density ratio; **(D)** mean of diameter of collagen; **(E)** stdev of average elastin intensity. The blue points indicated the dynamic changes of patients who achieved pCR in NAIT; the red points indicated the dynamic changes of patients who did not achieve pCR in NAIT. n_{pCR}=10, $n_{Non\text{-}pCR}$=16, n refers to the number of slices. Outliers were removed using the ROUT method with an aggressive Q=1%. If the data conforms to a Gaussian distribution, paired t-test was used to analyze the significance of dynamic changes of each factor pre- and post-treatment; otherwise, Wilcoxon matched-pairs signed rank test was applied. Significance levels are indicated as follows: *P < 0.05, **P < 0.01, ***P < 0.001, ****P < 0.0001.

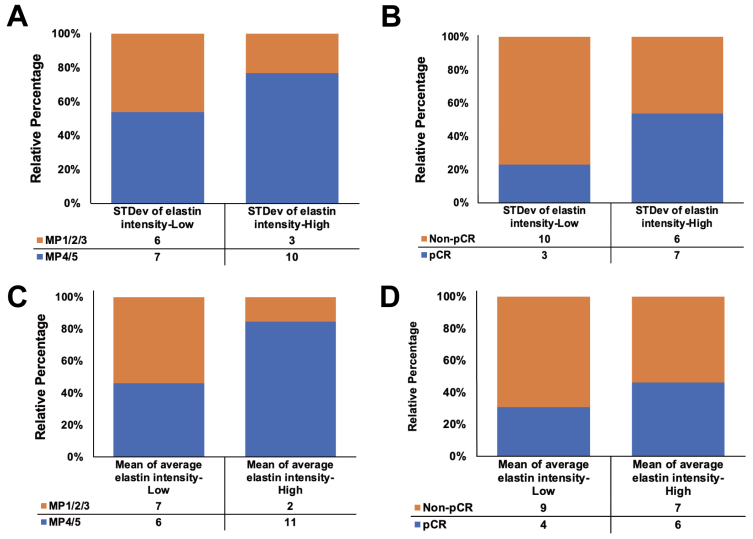

Supplementary Figure 7. Label-free MPM factors predict the efficacy of neoadjuvant immunotherapy. (A)&(B) Correlation analysis of the baseline level of stdev of elastin

intensity and responses of patients with breast cancer to NAIT. **(C)&(D)** Correlation analysis of the baseline level of the mean of average elastin intensity and responses of patients with breast cancer to NAIT. MP, Miller-Payne grading system; pCR, pathological complete response; Non-pCR, non-pathological complete response.
